# G4-QuadScreen: A Computational Tool for Identifying Multi-Target-Directed Anticancer Leads against G-Quadruplex DNA

**DOI:** 10.3390/cancers15153817

**Published:** 2023-07-27

**Authors:** Jyotsna Bhat-Ambure, Pravin Ambure, Eva Serrano-Candelas, Cristina Galiana-Roselló, Ariadna Gil-Martínez, Mario Guerrero, Margarita Martin, Jorge González-García, Enrique García-España, Rafael Gozalbes

**Affiliations:** 1 MolDrug AI Systems SL, c/Olimpia Arozena Torres, 46018 Valencia, Spain; jambure@moldrug.com; 2 ProtoQSAR SL, Centro Europeo de Empresas Innovadoras (CEEI), Parque Tecnológico de Valencia, 46980 Valencia, Spain; pambure@protoqsar.com (P.A.); eserrano@protoqsar.com (E.S.-C.); 3 Department of Inorganic Chemistry, Institute of Molecular Science, University of Valencia, 46980 Valencia, Spain; cristina.galiana@uv.es (C.G.-R.); ariadna.gil@uv.es (A.G.-M.); jorge.gonzalez@uv.es (J.G.-G.); enrique.garcia-es@uv.es (E.G.-E.); 4 Biochemistry and Molecular Biology Unit, Biomedicine Department, Faculty of Medicine and Health Sciences, University of Barcelona, 08036 Barcelona, Spain; mguerrero@ub.edu (M.G.); martin_andorra@ub.edu (M.M.); 5 Clinical and Experimental Respiratory Immunoallergy (IRCE), Institut d’Investigacions Biomediques August Pi i Sunyer (IDIBAPS), 08036 Barcelona, Spain

**Keywords:** cancer, G4 quadruplex, multi-tasking QSAR, virtual screening, molecular docking, drug design, FRET experiments, FID assays, MTT assays

## Abstract

**Simple Summary:**

G-quadruplexes (G4s) are guanine-rich, four-stranded nucleic acid structures that are abundantly found in the promoter region of various oncogenes (*cMYC*, *cKIT*, *KRAS*, etc.) and in the telomeric region. The ligand-induced stabilization of G4s is shown to be efficient in targeted cancer therapy, and simultaneously targeting multiple G4s is beneficial. Thus, this study aimed to achieve the ‘stabilization of G4s with multi-target directed ligands (MTDL)’. We have developed different multi-tasking QSAR models to predict G4 interaction, G4 stabilization, G4 selectivity, and cytotoxicity and we have implemented them in the first computational tool, ‘G4-QuadScreen’, derived from this robust methodology with the functionality to screen-out a library of small-ligand molecules against G4 DNAs. A virtual screening using this ‘G4-QuadScreen’ server and a posterior experimental validation has allowed us to identify a total of three compounds with strong inhibitory effect on various human cancer cell lines, demonstrating the usefulness of computational tools to accelerate the discovery of novel anticancer therapies.

**Abstract:**

The study presents ‘G4-QuadScreen’, a user-friendly computational tool for identifying MTDLs against G4s. Also, it offers a few hit MTDLs based on in silico and in vitro approaches. Multi-tasking QSAR models were developed using linear discriminant analysis and random forest machine learning techniques for predicting the responses of interest (G4 interaction, G4 stabilization, G4 selectivity, and cytotoxicity) considering the variations in the experimental conditions (e.g., G4 sequences, endpoints, cell lines, buffers, and assays). A virtual screening with G4-QuadScreen and molecular docking using YASARA (AutoDock-Vina) was performed. G4 activities were confirmed via FRET melting, FID, and cell viability assays. Validation metrics demonstrated the high discriminatory power and robustness of the models (the accuracy of all models is ~>90% for the training sets and ~>80% for the external sets). The experimental evaluations showed that ten screened MTDLs have the capacity to selectively stabilize multiple G4s. Three screened MTDLs induced a strong inhibitory effect on various human cancer cell lines. This pioneering computational study serves a tool to accelerate the search for new leads against G4s, reducing false positive outcomes in the early stages of drug discovery. The G4-QuadScreen tool is accessible on the ChemoPredictionSuite website.

## 1. Introduction

### 1.1. Cancer

According to the most recent report provided by GLOBOCAN 2020, the International Agency for Research on Cancer estimates that there will be approximately 21.9 million new cases of and 11.4 million deaths resulting from cancer worldwide by 2025 [1]. Although current anticancer treatments have significantly improved in recent times, many issues still need to be resolved, such as resistance development, efficiency, and toxic side effects. Cytotoxic agents are generally used in cancer therapy; however, their nonspecific modes of action lead to highly toxic side effects. The development of resistance is also a major hurdle. Thus, finding a more suitable, effective, and least toxic anticancer therapy is an urgent need.

Targeting proteins that are associated with various cancer biochemical pathways is accepted as an efficient alternative to overcome the inconveniences caused by cytotoxic agents. Nevertheless, the literature presents plenty of examples of drugs that show therapeutic efficacy against individual aberrant proteins involved in cancer end up losing their effectiveness due to the appearance of resistance [2,3,4,5]. This resistance can arise from secondary mutations in the target itself, the activation of adaptative loops, or the engagement of alternative oncogenic pathways.

As an alternative, direct targeting the transcription of the protein will help to overcome these resistance issues, especially those derived from the appearance of secondary mutations and the activation of adaptative loops. DNA transcription can be controlled by targeting the canonical secondary structure of DNA, which, despite being a more direct strategy, imposes a hurdle of non-selectivity, making the structure more prone to toxic side effects. Alternatively, the binding of ligands with the non-canonical DNA structures termed “G-quadruplexes” can resolve this non-selectivity issue in cancer therapies.

### 1.2. G-Quadruplex as a Potential Anticancer Target

In the guanine-rich DNA sequences form of the non-canonical G-quadruplex (G4) structure, four guanine bases form a planar tetrad through the Hoogsteen hydrogen-bonding pattern (Figure S1A). Multiple tetrads align over each other and are stabilized via π–π stacking interactions. Partial negative charges accumulated towards the central core of the tetrad because carbonyl oxygen atoms are further counterbalanced by coordination bonds formed with the channel metal ions (Figure S1B). These stacked tetrads are further interconnected by linking loop bases that generate grooves of different dimensions. Topological variations in G4 are offered by various arrangements of connecting loop bases and orientations of guanine strands, denoting the high polymorphic nature of G4s depending on the sequence and conditions (Figure S1C). Thus, these higher-order globular structures of DNA can offer adequate and selective binding sites equivalent to protein targets [6].

Guanine-rich, single-stranded DNA is present at the telomeric end of the chromosome; it is non-replicative and becomes shorter with each cell cycle. Once the length of the telomeric region is shortened beyond a certain threshold, it initiates cell cycle arrest and cell death. However, in cancer cells, telomerase binds and maintains the length of the telomeric end, thus leading to immortality of the cells. The ligand-induced stabilization of G4 at telomeric DNA impairs the binding of telomerase, thus recovering the normal shortening of telomeric DNA; then, further normal cell cycle arrest and cell death occur [7] (Figure S2A). Also, G4-forming motifs are abundantly present in the promoter region of various oncogenes (*cMYC, cKIT, KRAS, BCL2,* etc.), and the dynamic structure of G4 regulates the expression of these oncogenes (Figure S2B). Oncogenes play a crucial role in cell proliferation, cell differentiation, and cell death and are overexpressed in cancer. The ligand-induced stabilization of respective G4s can downsize this overexpression, further bringing the functioning of the cell cycle to a normal state. Thus, telomeric G4 and G4 motifs from the promoter regions of various oncogenes are considered significant targets in cancer therapy [8].

Generally, G4 ligands share some chemical features, such as planar aromatic rings, to form the crucial π–π stacking interactions with guanine planes, as well as positively charged groups to form the electrostatic interactions with negatively charged backbone phosphate groups [6,9,10,11,12,13]. Though these features facilitate great G4 binding capacity, they exert poor cell permeability, which is their main biopharmaceutical limitation [14]. Thus, it is necessary to find an adequate balance between the G4 binding capacity and druggability of the lead molecules. Also, the selectivity of ligands towards G4s over the duplex DNA is a critical factor for avoiding off-target activities and normal cell toxicities. In the current study, while a virtual screening, we have paid keen attention to the drug-likeness of the compounds and their selectivity towards G4s over duplex DNAs.

### 1.3. Multi-Target Drug Designing

For the treatment of complex diseases (such as cancer, multiple sclerosis, Alzheimer’s disease, etc.), drugs acting on a single-target enzyme or receptor are often found insufficient. Multi-target drug design is an emerging rational approach that focuses on the development of drug candidates that can simultaneously act on multiple targets [15]. In this context, various oncogenes playing key roles in cell cycle functioning are deregulated and overexpressed in various types of cancers. Table 1 lists some of the oncogenes whose expression is regulated by the presence of G4s within their promoter regions (their roles in the cell cycle and a few of the associated cancer conditions are also enlisted).

Thus, for the efficient treatment of complex and multifactorial diseases like cancer, the current study focuses on multi-target directed ligands (MTDL) that can simultaneously target multiple (two or more) G4 motives located in the promoter regions of respective oncogenes and/or telomeric regions.

### 1.4. Multi-Target, Multi-Tasking QSAR Modeling

Quantitative structure–activity relationship (QSAR) modeling is a widely used computational technique to develop a quantitative relationship between the descriptors representing chemical features and the activity/property variable for a series of compounds. The resulting models can then be used to predict the behavior of other compounds for which the descriptors are easy to calculate. In the present scenario, the QSAR models that are already reported in the literature are limited to individual G4 targets [16,17,18,19], and thus the studied compounds or leads might have activity against respective individual oncogenes. With regard to quadruplexes, G4 studies have a vast number of variables; the activity of one molecule is defined by many factors such as the type of oncogene, sequence of the DNA, buffer conditions, type of cell lines, type of assay, etc. The classical QSAR approach can only accommodate one single experimental condition at a time; therefore, it cannot be applied in addressing data related to G4.

In the current study, multi-target QSAR (mtQSAR) models were developed to identify potential MTDLs for different types of human G4s. At the initial stages of the usual drug discovery process, G4 ligands were evaluated based on their capacity to interact with G4, their capacity to stabilize G4, their selectivity towards G4 over duplex DNAs, and their cellular activity. All these aspects of evaluation were taken into consideration in these mtQSAR models. Instead of classical QSAR, a multi-tasking QSAR approach was adopted in the development of mtQSAR models. The Box–Jenkins moving average approach [20,21] was employed; using this approach, compounds cannot only be merged with response data determined in diverse experimental conditions but also derive a mtQSAR model by employing multiple biological responses against different G4 targets.

### 1.5. Aim of the Study

The goal of this study is to provide an easy solution towards identifying potential small lead molecules against human G4 DNA structures from various gene areas. We introduce ‘G4-QuadScreen’, a user-friendly, web-based computational tool for identifying MTDLs against G4s. The mtQSAR models developed in the study were compiled together and used as a knowledge base in ‘G4-QuadScreen’. This tool facilitates the screening of a library of molecules against G4-forming motifs belonging to telomeres (*hTel*) and four oncogenes: *cMYC, KRAS, cKIT1,* and *cKIT2*. Also, it evaluates four properties of ligands such as G4 binding, G4 stabilization, G4 selectivity, and cytotoxicity.

Virtual screening was performed with the help of ‘G4-QuadScreen’ as well as molecular docking. Selected chemicals from the screened MTDLs were further evaluated using FRET melting experiments. Then, top hits from the analysis of the FRET melting values were further evaluated using a TO displacement assay and cell-based assays.

This study introduces several novel steps. First, from a methodological point of view, completely novel mtQSAR models were developed in this study, thus going beyond the traditional molecular simulations of individual endpoints. Secondly, these models were made accessible for virtual screening purposes in a completely new web-based platform, ‘G4-QuadScreen’, integrating a collection of QSAR predictive models focused on the anticancer potential of chemicals. This study also offers a robust protocol of how to collectively utilize various machine learning approaches and molecular modeling tools in the early stages of drug discovery, which can be implemented in tackling other complex diseases. Finally, the originality of this study derives from the fact that this is the first in-depth computational study to identify novel potential compounds that can stabilize multiple G4s simultaneously and become leads in cancer treatment.

## 2. Materials and Methods

### 2.1. Dataset Collection and Curation

#### 2.1.1. Dataset Collection

**Figure 1:**
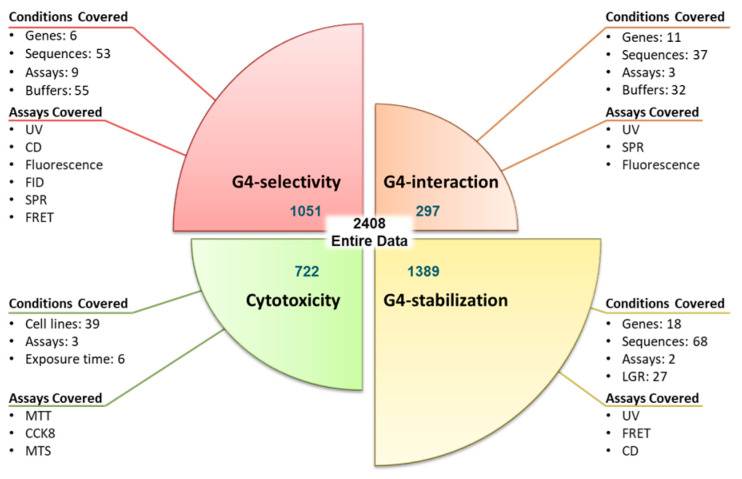
Distribution of data: complete data of 2485 datapoints are distributed for developing four models, e.g., G4 selectivity (1722 datapoints), G4 interaction (202 datapoints), G4 stabilization (1301 datapoints), and cytotoxicity (982 datapoints). Assays covered in each model are highlighted in cyan-colored boxes. Conditions covered in each model are enlisted in each section. These number of datapoints are arrived after data curation; the original data collected were higher in number.

The data were collected from the G4 ligand database, G4LDB (https://www.g4ldb.com/, accessed on 30 September 2021), and each datapoint was re-confirmed from the respective scientific study [22,23]. Initial raw data comprised 2485 datapoints with available activity information of ligands for several G4s. These datapoints represent the G4 interaction, G4 stabilization, G4 selectivity, and cytotoxicity of these G4 ligands determined in different experimental protocols and conditions. The distribution of data as per the evaluation criteria and information regarding respective assays and experimental conditions is illustrated in Figure 1. The data of various experimental assays and their contribution towards each evaluation criteria of the concerned models are shown in Table 2.

#### 2.1.2. Data Curation

**Figure 2:**
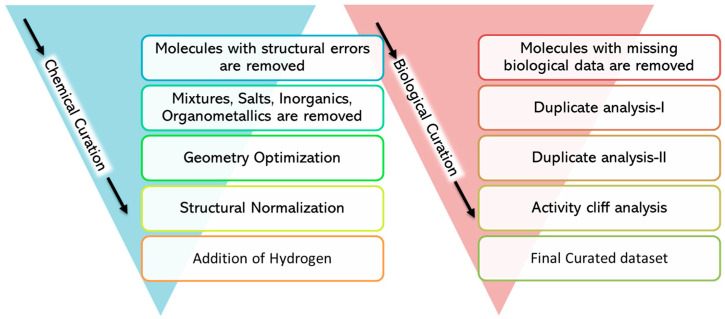
Steps involved in ‘chemical data curation’ followed by the steps involved in ‘biological data curation’.

Data curation is a crucial task, especially while handling “big data”. We followed the protocol outlined by the guidelines offered by QSAR experts [24,25]. The quality of the data was analyzed for both chemical as well as biological aspects. The steps we followed for the curation of the data are illustrated in Figure 2.

The curation of the chemical data was performed using the KNIME workflow developed by Ambure and collaborators [26]. The curation of chemical data includes various steps, such as checking and rectifying the errors in the chemical structure, the exclusive handling of inorganic/organometallic/salts, the normalization of the chemical structures, and, finally, the addition of explicit hydrogens atoms.

The curation of biological data was performed using an in-house Python script. This script can handle big data in standard, single experimental condition as well as complex data with multiple experimental conditions (the same as the data used in the current study). Firstly, data with missing endpoints were removed, and then a duplicate analysis was performed in two steps. In the “duplicate analysis I” step, the datapoints were considered duplicates only if they were exactly identical in the structure, all experimental conditions, as well as if the endpoint values matched among them. Then, only one of the duplicates was kept, and the other datapoints were removed. In the “Duplicate analysis II” step, if the experimental conditions and structure were exactly the same but the endpoint values were slightly different (difference less than 0.5), the average of the endpoint values was considered and assigned to one of the duplicates, and the rest of the duplicate data were removed. However, if duplicates had a difference in the endpoint values of more than 0.5, they were processed cautiously. In such cases, if all the endpoint values placed the data into a similar category (based on the activity threshold of the respective model), e.g., active or inactive, then one datapoint was kept and the other datapoints were removed. However, if they were classified differently, then all such datapoints were removed. In studies of G4 ligands, endpoint values are highly dependent on each experimental condition and changing just one experimental condition can drastically affect the endpoint value; thus, though activity cliff analysis is a major part of biological data curation, it was not used in the current study.

### 2.2. Descriptor Calculation and Data Preprocessing

Using an in-house Python script, 12,810 PaDEL descriptors (including fingerprints) [27] and 4776 other structural descriptors were calculated. The constant and highly inter-correlated descriptors were removed with a variance cut-off of 0.0001 and a correlation coefficient cut-off of 0.99 using the V-WSP data pretreatment tool (DPT) [28]. Further data with missing descriptors were removed using an in-house Python script.

### 2.3. Model Development and Validation

Four multitasking mtQSAR models, which can predict G4 selectivity, G4 interaction, G4 stabilization, and cytotoxicity, were developed. The Box–Jenkins moving average approach was employed to calculate modified descriptors that integrate structural information with experimental conditions. The experimental conditions incorporated in each model are illustrated in Figure 1. In the case of G4, the topology (thus ligand binding) is defined by buffer conditions and the DNA sequence under consideration. Thus, in three of the models, ‘G4-Selectivity, G4-Interaction, and G4-Stabilization’, buffer conditions and sequence information are enforced (Figure 1). In the case of the cytotoxicity model, the type of cell line indicates the type of cancer; thus, the prediction of the model can assist in inferring if the G4 ligand is effective against a particular type of cancer. Exposure time definitely has an impact on the IC_50_ values. Thus, these two experimental conditions are enforced in the cytotoxicity model. The classes (positive = 1 and negative = 0) were assigned according to the predefined cut-off values, as shown in Table 3.

**Figure 3:**
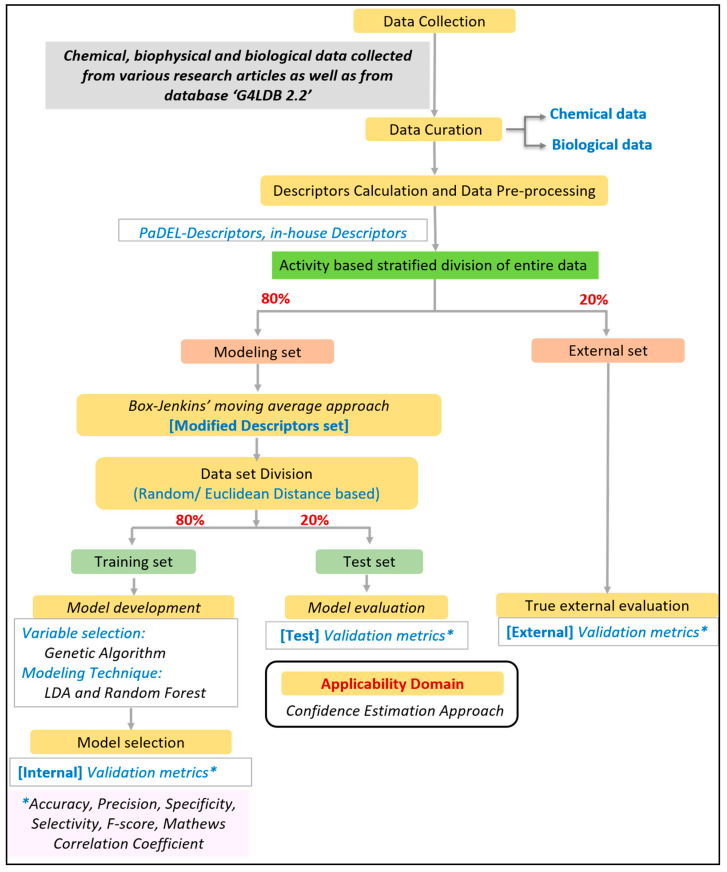
Steps followed in multi-tasking mtQSAR modeling. * Validation metrics refer to accuracy, precision, specificity, selectivity, F-score and Matthews correlation coefficient (MCC).

All tasks related to the development of the mt-QSAR model were performed using QSAR-Co software (v. 1.1.0) [29]. The modified descriptor set was based on the Box–Jenkins moving average approach. Further steps involved dataset division, variable selection, model development, validation, and the determination of the applicability domain. The tasks performed right from the dataset collection to model development are listed in Figure 3.

In this study, the dataset was divided into a modeling set (80% of the entire data) and an external set (20% of the entire data) with an activity-based stratified division approach. In the calculation of modified descriptors, the information from both training and test sets were utilized, provoking a data leakage to a small extent. Thus, one external set was kept aside for validation purposes, which was completely untouched while training the model. Furthermore, modified descriptors were calculated and processed in the modeling. The modified modeling set was divided into a training set (80%) and a test set (20%) using the random approach (except in the case of the G4 interaction model, where data was divided based on the Euclidean-distance-based similarity approach). Both division approaches are achievable in QSAR-Co software (v. 1.1.0). The training set was employed for the development and selection of the optimal model, whereas the test set was exclusively utilized to validate it. The genetic algorithm (GA) was used as a variable/feature selection technique. The final mtQSAR models were developed using two machine learning techniques, namely linear discriminant analysis (LDA) and random forest (RF), which were implemented from QSAR-Co with default parameters [30,31,32]. Firstly, GA-LDA was run to check the most contributing descriptors, and both Mathew’s correlation coefficient (MCC) and Wilks lambda (λ) parameter [33] were employed to compute the fitness score in the GA. Based on the fitness score, the best model was selected in each generation. Top descriptors were selected based on the results of the GA-LDA (e.g., from the model with good fitness scores), which were then utilized to derive an RF model. Parameters for RF were optimized to obtain the best internal validation results. The optimal LDA and RF models were evaluated and selected on the basis of qualitative validation metrics computed for the training set, and then the selected models were externally validated using the test set. The models generated in QSAR-Co were remodeled with the LDA and RF machine learning methods implemented in scikit-learn (version 0.24.2) since the final screening tool ‘G4-QuadScreen’ was built with Python and scikit-learn functionalities. The modeling parameters used for each model are listed in Table S1.

### 2.4. Applicability Domain

Three different approaches to estimate the applicability domain (AD) of the QSAR models were implemented: (i) The first is based on the structural similarity of the compound to that present in the training set. MACCS fingerprints were used to define the structure and similarity based on the Tanimoto distance. (ii) The second is a distance-based method using Euclidean distance. (iii) The third is also a distance-based method that uses the Leverage approach [34,35]. One compound is considered to be inside the AD if it fits at least one of the three methods.

### 2.5. G4-QuadScreen Web-Based Computational Tool

The web-based G4-QuadScreen application was developed using Python as a back-end language and a Django framework as a front-end language.

### 2.6. Virtual Screening and Molecular Docking

#### 2.6.1. Virtual Screening

**Figure 4:**
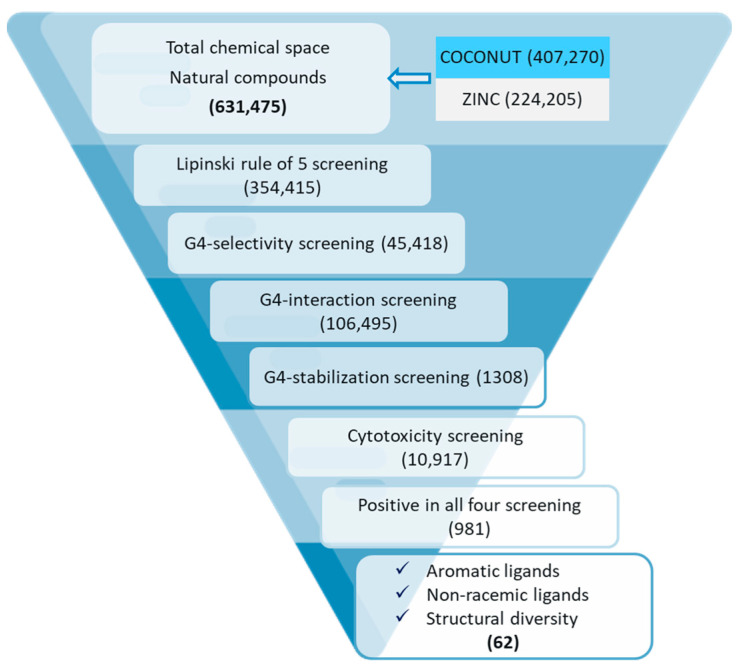
Stepwise workflow followed by the virtual screening of natural compounds to find possible G4 ligands.

A library of 631,475 natural compounds was obtained from ligand databases, namely ZINC [36] and COCONUT [37]. The curation of the chemical data of the library was conducted using the same protocol followed for the modeling part (refer to Section 2.1.2). With the help of an in-house KNIME workflow, curated data were further passed through the criteria of Lipinski’s rule of 5, and 354,415 compounds passed the criteria. These compounds were further screened using four multitasking mtQSAR models, and 981 of them were predicted as positive in all models. Among these 981 molecules, 62 molecules were selected for further study with the following selection criteria: (i) molecules have aromatic and or planar rings, (ii) they are non-racemic, and (iii) molecules are structurally diverse. The complete workflow followed by virtual screening is illustrated in Figure 4.

#### 2.6.2. Molecular Docking

All 62 molecules were docked against G4 structures of telomere/oncogenes, viz., *hTel, cMYC, cKIT1, cKIT2,* and *KRAS*; the details of the used PDB files are listed in Table S2. The pKa of ionizable groups within selected molecules was estimated using the graph-convolutional neural network provided by the web server ‘MolGpKa’ [38]. Charges over ionizable groups at a pH value of 7.4 (experimental condition) were determined using the predicted pKa values with the formula:

Charge over acid group = (−1) × (α)(1)

Charge over base group = (+1) × (1 − α)(2)

(3) $$\alpha \;(\mathrm{degree}\;\mathrm{of}\;\mathrm{dissociation})=\frac{1}{10^{\left(pKa-pH\right)}+1}$$

Molecular docking was performed using AutoDock Vina [39] as implemented in YASARA [40]. The simulation cell was built at a 0.3 Å distance around all the atoms of the receptors. The designed functionality ‘dock_runscreening’ using the standard values of the macro file (AMBER03 force field with rigid receptor and flexible ligand) was employed to dock the selected compounds.

### 2.7. FRET Melting Experiments of Screened Compounds

The 62 molecules were purchased from Molport and used without further purification. The DNA oligonucleotides were purchased from IDT (Integrated DNA Technologies, Belgium) and were of HPLC purity grade. Labeled DNA was dissolved as a 20 µM stock solution in MilliQ water, annealed with a 400 nM concentration in potassium cacodylate buffer (10 mM KCl, 90 mM LiCl, 10 mM LiCac, pH 7.3) at 90 °C for 10 min, and then slowly cooled to room temperature overnight. Ligands were dissolved from stock solutions to final concentrations in the buffer. Each well of a 96-well plate (Applied Biosystem, Waltham, MA, USA) was prepared with 60 µL, with a final 200 nM DNA concentration and two concentrations of tested ligands (2 µM and 4 µM). Measurements were performed on a PCR AriaMx (Agilent Technologies, Santa Clara, CA, USA) with excitation at 450–495 nm and detection at 515–545 nm. Readings were taken from 25 °C to 95 °C at intervals of 0.5 °C, maintaining a constant temperature for 30 s before each reading. Each measurement was carried out in triplicate. The normalized fluorescence signal was plotted against the compound concentration, and the ΔT_m_ values were determined.

### 2.8. TO Displacement (FID) Assay of Selected Compounds

The top ten hits from the analysis of the FRET melting values were further evaluated using a TO displacement assay and cell-based assays.

The TO assay follows the decrease in the fluorescence emission of the thiazole orange (TO) upon the ligand-induced displacement of TO from the DNA-TO adduct. Measurements were performed on a Varian Cary Eclipse Spectrometer following the protocol reported by Teulade-Fichou’s team [41]. Oligonucleotides were prepared via heating at 90 °C in LiCaco buffer (100 mM KCl, 10 mM LiCaco pH 7.2), and then slowly cooled to room temperature overnight. Oligonucleotide structures were formed at a 250 μM strand concentration. The test was designed as follows: a mixture of pre-folded quadruplex (1 μM) and TO (2 μM), in LiCaco buffer (100 mM KCl, 10 mM LiCaco pH 7.2), was titrated with an increasing amount of ligand (from 0.25 to 20 equiv.), in which a 2 min equilibration period elapsed before the fluorescence spectrum was recorded. The fluorescence area (FA, 510–850 nm) was converted into a percentage displacement (PD) using the following formula:

(4) PD=100−FAFA0×100

FA_0_ is FA before the addition of a ligand.

### 2.9. Cell-Based Assays of Selective Compounds

#### 2.9.1. Cell Culture

Cervical (HeLa), breast (MCF-7), and lung (A549) cancer cell lines were provided by the Central Service for Experimental Research (SCSIE) at the University of Valencia. The cells were cultured in Dulbecco’s Modified Eagle Medium (DMEM) with 4.5 g/L glucose (Gibco, Waltham, MA, USA), supplemented with penicillin (100 U/mL) plus streptomycin (100 µg/mL) (Gibco) and 10% fetal bovine serum (FBS), using standard cultivation conditions (37 °C, 5% CO_2_). Cells were kept continuously under confluence before splitting twice a week. The possibility of contamination was excluded by performing regular mycoplasma tests.

Human GIST cell lines were kindly provided by Dr. S. Bauer (University Duisburg-Essen, Medical School, Essen, Germany). Imatinib-sensitive GIST-T1 (KIT mutation exon 11 Val560_Tyr578del) cells were cultured in IMDM media supplemented with 15% FBS, 1% L-glutamine, 50 U/mL penicillin, and streptomycin [42]. GIST430/654 (KIT mutation Exon 11 Val560_Leu576del, exon 13 Val654Ala) cells were cultured in IMDM media supplemented with 15% FBS, 1% L-glutamine, 50 U/mL penicillin and streptomycin, and an additional 200 nM imatinib mesylate (Sigma-Aldrich, St. Louis, MO, USA) to maintain selective pressure [43]. The mycoplasma test was routinely performed in all cell lines used.

#### 2.9.2. Cell Viability Assay

HeLa, MCF-7, and A549 cancer cells were seeded at a density of 5000 cells/well and maintained in an incubator overnight at 37 °C with 5% CO_2_. The compounds were suspended in a medium at final concentrations of 100 mg/mL in DMSO and analyzed in a decreasing dose curve from 50 to 100 μM. As a control, cells were treated with 1–2% DMSO. The number of viable cells in the culture was determined via the quantification of ATP, using the Cell Titer-Glo luminescent assay kit (Promega, Madison, WI, USA). Following the manufacturer’s instructions, the cells were plated in 96-well plates and treated 24 h later with the compounds for 48 h and concentrations, followed by the addition of a Cell Titer-Glo reagent. Luminescence was detected using a multi-well Synergy Mx scanning spectrophotometer (Bio-Tek, Winooski, VT, USA).

GIST cell lines were seeded in 96-well plates (10,000 cells/well) and treated with the compounds for the indicated concentrations. Cell viability was measured using the colorimetric WST-1 assay (Roche™ Diagnostics, Mannheim, Germany) upon 72 h of treatment according to the manufacturer’s protocol. Data were expressed as the mean ± standard deviation (mean ± SD) from three independent experiments.

## 3. Results and Discussion

### 3.1. G4 Selectivity Model

Among the developed LDA and RF models, the RF model was selected as the best one since its validation parameters were better. The optimal values obtained for statistical parameters such as accuracy, precision, sensitivity, specificity, F-measure, and Mathew’s correlation coefficient (MCC) are indicative of the good discriminatory power of the RF model (refer to Table 4). The statistical parameters were further obtained for 10-fold cross validation, test set, external set, and external set compounds within the applicability domain of the model. Except for decreased specificity, the performance of the model is satisfactory in all the sets. Thus, it can be concluded that the RF mtQSAR model can differentiate between selective and non-selective G4 ligands.

The model comprises fifteen descriptors combined with four experimental conditions: gene sequence, type of buffer, type of assay, and type of oncogene. In Table S3, the meaning, importance of each feature, corresponding experimental condition, source, and type of each descriptor are summarized.

### 3.2. G4 Interaction Model

Among the developed LDA and RF models, the LDA model was selected as the best one as the validation parameters were better (refer to Table 5). The optimal values obtained for statistical parameters support the good discriminatory power of the model. The statistical parameters obtained for 10-fold cross validation, test set, external set, and external set compounds within the applicability domain of the model further indicate that the performance of the model is acceptable in all the sets. Thus, it can be concluded that the developed LDA mtQSAR model is able to differentiate between G4 binders and non-binders.

The model is comprised of ten descriptors combined with three experimental conditions, viz., gene sequence, type of buffer, and type of oncogene. In Table S4, the meaning, LDA coefficient, corresponding experimental condition, source, and type of each descriptor are summarized.

### 3.3. G4 Stabilization Model

Among the LDA and RF models developed, the RF model was selected as the best one (validation parameters are shown in Table 6). The optimal values obtained for statistical parameters support the good discriminatory power of the developed RF model. The statistical parameters obtained for the 10-fold cross validation, test set, external set, and external set compounds within the applicability domain of the model further indicate that the performance of the model is acceptable in all the sets. Thus, it can be concluded that the developed RF mtQSAR model is aptly capable of differentiating between G4 stabilizers and non-stabilizers.

The model comprises ten descriptors combined with five experimental conditions: ligand to G4 ratio, gene sequence, type of buffer, type of assay, and type of oncogene. In Table S5, the meaning, feature importance, corresponding experimental condition, source, and type of each descriptor are summarized.

### 3.4. Cytotoxicity Model

The RF model was also selected when considering the validation parameters (refer to Table 7). The optimal values obtained for the statistical parameters support the good discriminatory power of this model. The statistical parameters obtained for the 10-fold cross validation, test set, external set, and external set compounds within the applicability domain of the model further indicate that the performance of the model is acceptable in all the sets. Thus, it can be concluded that the RF mtQSAR model is able to differentiate between cytotoxic and non-cytotoxic ligands.

The model comprises nine descriptors combined with three experimental conditions: exposure time, type of cell line, and type of assay. In Table S6, the meaning, feature importance, corresponding experimental condition, source, and type of each descriptor are summarized.

### 3.5. G4-QuadScreen Web-Based Computational Tool

As shown, the four models were found to be robust and have good discriminatory power. Subsequently, these models were deployed in the form of a user-friendly web-based computational tool, viz. ‘G4-QuadScreen’.

Though the models were built incorporating numerous experimental conditions and numerous oncogene G4 targets (refer to Figure 1), in the final deployed tool, screening is offered against the specific G4 targets and specific experimental conditions listed in Table 8. The specific experimental conditions and G4 targets selected for the screening module are based on their abundance in the modeling data; that way, the offered activity predictions are more reliable. Also, some conditions are selected as they were found to be majorly used by G4 researchers in the laboratory.

**Figure 5:**
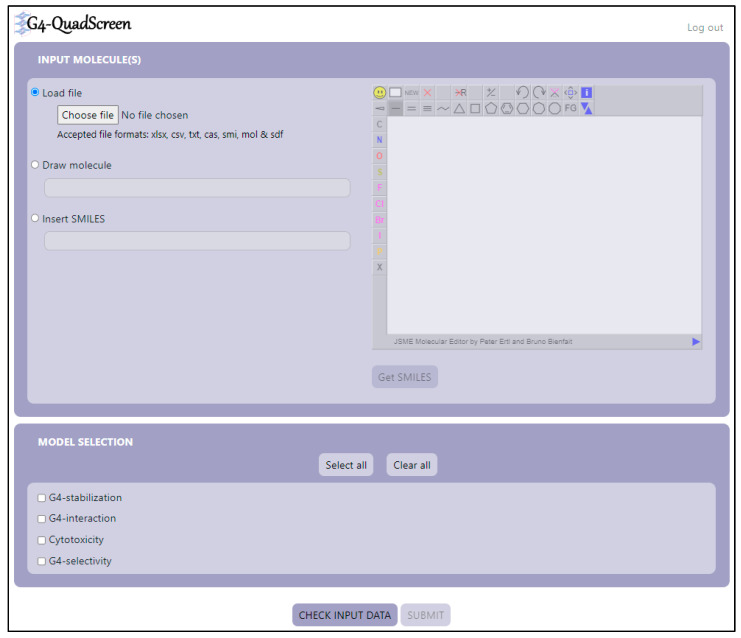
Graphical user interface of a web-based application, ‘G4-QuadScreen’.

**Figure 6:**
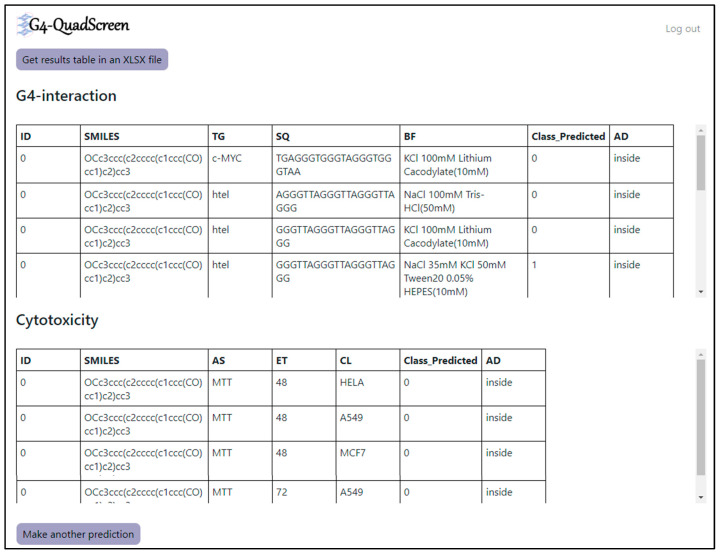
Example of the output of the prediction by G4-QuadScreen.

‘G4-QuadScreen’ (see Figure 5) works in a systematic way, which makes it extremely user-friendly and efficient. It predicts the essential G4-oriented properties such as G4 selectivity, G4 interaction, G4 stabilization, and cytotoxicity for an input molecule. The first node, “INPUT MOLECULES”, offers users three ways to provide information about molecules. Firstly, the user can browse and read the input file with a list of molecules in a SMILES format; it accepts files in various forms. Secondly, the user can draw a 2D chemical structure of interest and fetch the SMILES for the same model using the tab “Get SMILES”. Thirdly, for an individual molecule, the user can just type SMILES in a text field. The second node, “MODEL SELECTION”, offers a checklist, where the user can select which G4 property needs to be evaluated for their ligand of interest. The “CHECK INPUT DATA” tab scrutinizes if there are any errors (e.g., disconnected structures, mixtures, big molecules, etc.) in the input SMILES. After the verification of the input data, the user can “SUBMIT” the job. This tool offers inbuilt functionality for calculating the descriptors needed for predicting the respective properties. Output is in the form of prediction matrices for G4 properties selected in a “MODEL SELECTION” node. Each table contains the SMILES notation of the input molecule, experimental conditions, activity prediction for those specific conditions, and the denotation of whether the query molecule is inside or outside of the applicability domain of the respective G4 model (refer to Figure 6). Also, the user can fetch the predictions in the form of an Excel file by the tab “Get results table in an XLSX file”. Thus, the tool is extremely functional; anyone can use it without any prior training.

### 3.6. Molecular Docking

**Figure 7:**
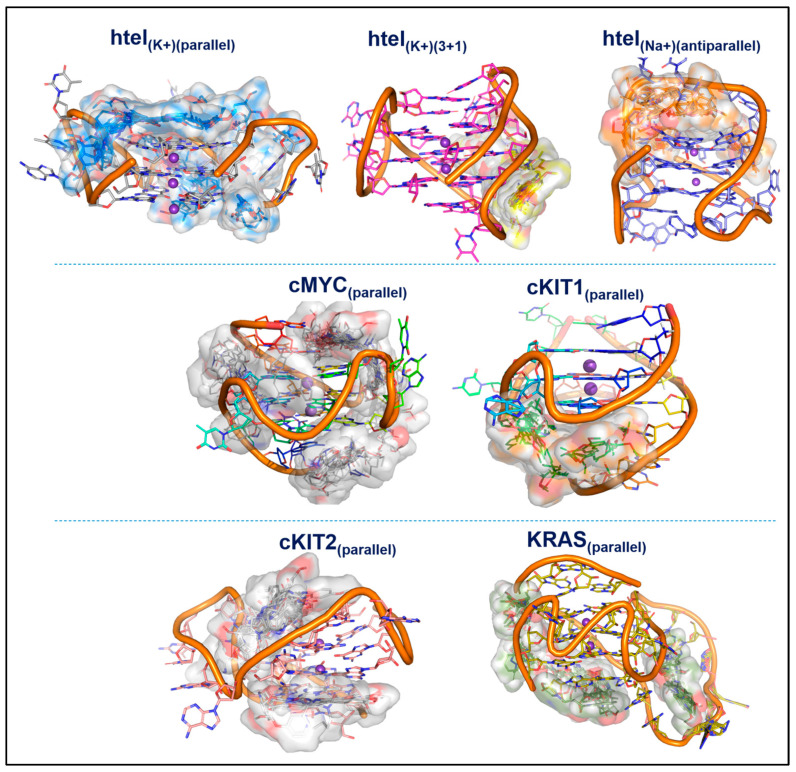
Ensembled docked poses of 62 ligands with their respective G4 domains; binding is highlighted with a semi-transparent surface.

The molecular docking results suggest that all 62 ligands bind to DNA motifs with similar binding energies; the average binding energies (kcal/mol) of 62 ligands and their binding sites are listed in Table 9. The ensembles of the docked poses of the 62 ligands in each G4 are illustrated in Figure 7.

When the G4 interaction screening results were compared with the docking results, it was observed that the 62 molecules seemed to be active against hTel (antiparallel) in screening and have good docking scores. However, in other G4 domains, docking failed to distinguish between active and inactive molecules (refer to Figure S3). Details of the binding energies of each ligand and binding residue with respective DNA motifs are provided in the supplementary information (Supplimentary_Docking-results.xlsx).

### 3.7. FRET Melting Experiments of Screened Compounds

**Figure 8:**
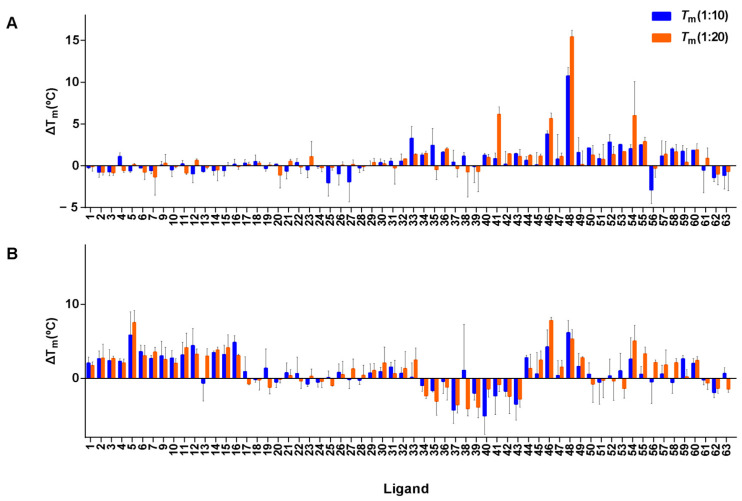
Representation of FRET melting values (ΔT_m_) for the interaction between the ligands and hTel (**A**) and cMYC (**B**) G4s. The concentration of DNA was 0.2 μM, and the [DNA]/[Ligand] ratios were 1:10 and 1:20. Errors denote the standard deviations of at least three independent experiments.

An initial screening experiment was conducted by FRET melting experiments to assess the stabilization and selectivity effect of the ligands with the G4 DNAs. The G4-forming sequences found in the promoter region of cMYC and cKIT2, telomeric region hTel, and the ds26 as a duplex DNA were taken into consideration (see Table S7 for nucleic acid sequences, topology, and genome localization). The threshold of ΔT_m_ > 4 °C was assigned to consider the ligand as a G4 stabilizer. The ligands, Lig-41, Lig-46, and Lig-54, stabilized hTel G4 at a higher ligand concentration (4 µM) (see Figure 8A); the strongest stabilization was seen with Lig-48 at both the ligand concentrations. With regard to cMYC G4, the ligands Lig-5, Lig-11, Lig-12, Lig-15, Lig-16, Lig-46, Lig-48, and Lig-54 showed the highest stabilization effect among all the ligands (Figure 8B). Lig-5, Lig-46, and Lig-48 showed the highest cMYC G4 stabilization. Interestingly, the ligands Lig-46, Lig-48, and Lig-54 were identified as G4 stabilizers for both cMYC and hTel G4s, suggesting that these ligands are multi-targeted G4 stabilizers. The stabilization effect of the ligand over ds26 is illustrated in Figure S4. The average ΔT_m_ observed for ds26 was <2 °C, thus supporting the selectivity of the screened ligands.

**Figure 9:**
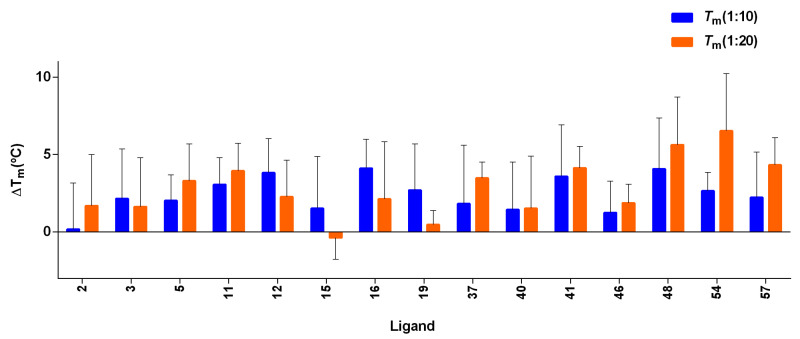
Representation of FRET melting values (ΔT_m_) for the interaction between the ligands and cKIT2. The concentration of DNA was 0.2 μM, and the [DNA]/[Ligand] ratios were 1:10 and 1:20. Errors denote the standard deviations of at least three independent experiments.

Furthermore, we investigated the G4 stabilizers found for hTel and cMYC against the G4-forming sequence in the promoter region of cKIT2 using FRET melting experiments (refer to Figure 9). Additionally, we analyzed Lig-57 against cKIT2 as it was classified as a cKIT2-stabilizer using the G4 stabilization model. According to the set threshold, ligands Lig-16, Lig-41, Lig-48, Lig-54, and Lig-57 stabilized this G4 significantly.

### 3.8. FID-TO Displacement Assay of Selective Compounds

To investigate the binding capacity of ligands showing G4 stabilization in FRET melting experiments (Lig-5, Lig-11, Lig-12, Lig-15, Lig-16, Lig-41, Lig-46, Lig-48, Lig-54 and Lig-57), fluorescence indicator displacement (FID) assays were performed with hTel and cMYC G4s. All of these ligands showed a low TO displacement, hampering our calculations of DC_50_ values from these titrations. Therefore, the percentage of displacement at the highest concentration used for each ligand is taken as a comparative measure, which is listed in Table S8. The largest TO displacement in cMYC corresponds to the ligand Lig-5 followed by Lig-48, whereas in hTel, Lig-46 shows the highest TO displacement but at a high ligand concentration.

Though all 10 ligands showed adequate G4 stabilization in the FRET melting experiments, the weak displacement of TO with the addition of the ligands was observed in FID assays. Thus, the binding poses of these ligands obtained via molecular docking against *cMYC* and *hTel* were thoroughly scrutinized. FID assays were conducted in a potassium buffer and *hTel* G4 attains ‘3 *+* 1 hybrid’ and ’parallel’ topologies in potassium; thus, both topologies of *hTel* were considered in the analysis of molecular docking. As shown in Table S9, the best docked poses show that ligands were majorly binding in the groove region and partially stacking to ends. Also, when an ensemble of other feasible docked poses of each ligand was analyzed (refer to Supplimentary_Docking-results.xlsx), it was found that the percentage of ligands solely interacting through end stacking was lower compared to that of groove binding and partial end stacking. The poor displacement of TO in the FID assays reflects that the interaction of the ligands occurred in different modes than end stacking.

### 3.9. Cell-Based Assays of Selective Compounds

Once we investigated the binding of the ligands to G4s, we assessed the cell viability of the ligands, Lig-5, Lig-11, Lig-12, Lig-15, Lig-16, Lig-41, Lig-46, Lig-48, Lig-54, and Lig-57, in cancer cell lines. We used HeLa (overexpress cMYC), MCF-7 (overexpress cMYC), A549 (overexpress cMYC), GIST-T1 (overexpress cKIT), and GIST430/654 (overexpress cKIT) derived from cervical, breast, lung, and gastrointestinal stromal cancers, respectively [44].

**Figure 10:**
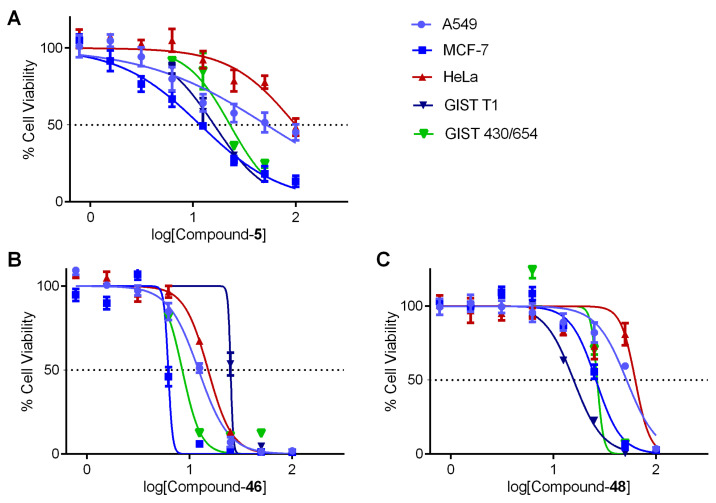
Dose–response curves of the lead compounds: Lig-5 (**A**), Lig-46 (**B**), and Lig-48 (**C**).

**Figure 11:**
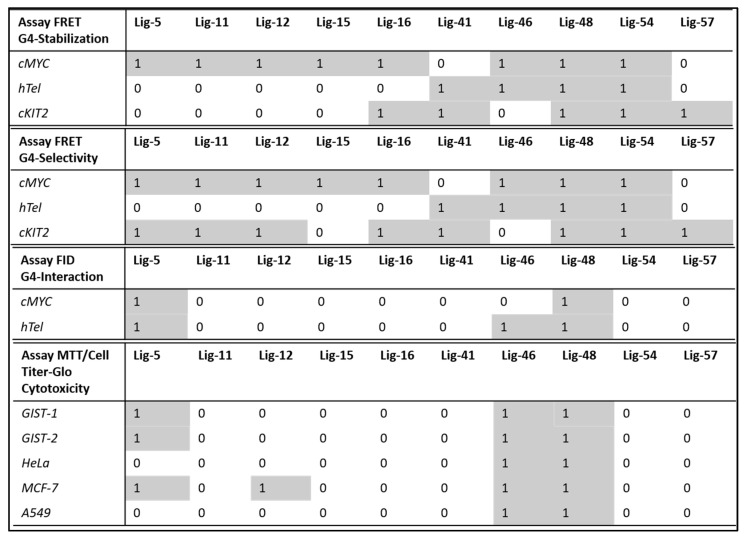
Summary of the G4 stabilization and G4 selectivity over duplex DNA derived by FRET experiments and G4 interaction capacity derived by FID assay and cytotoxicity observed against 5 cancer cell lines. The positive activity is denoted as 1 and highlighted in grey, and the inactive status is denoted as 0.

Among the tested ligands, Lig-46 and Lig-48 have a strong inhibition effect on all the cancer cell lines, making Lig-46 the most cytotoxic (Figures S5 and S6). We calculated the IC_50_ values for the ligands Lig-5, Lig-46, and Lig-48 (Figure 10), which are gathered in Table 10. These findings are in agreement with the previous results of FRET melting experiments because both Lig-46 and Lig-48 showed the highest stabilization effect on cMYC G4 and HeLa; MCF-7 and A549 overexpress these oncogenes. Moreover, Lig-46 and Lig-48 had a strong cytotoxic effect on GIST-T1 and GIST430/654, which agrees with the high cKIT G4 stabilization discovered via FRET experiments and cKIT overexpression in these cancer cell lines. Interestingly, Lig-48 yielded a larger stabilization effect on cKIT G4 and lower IC_50_ values in GIST cancer cell lines than Lig-46, suggesting a mechanism involving cKIT. Thus, according to our results, Lig-46 and Lig-48 can be assigned as multi target ligands because of the high G4 interaction and cytotoxicity in both hTel, cMYC, and cKIT G4s (see Figure 11). Also, Lig-5 showed activity against G4s and in three types of cancer cell lines. These three MTDLs can be explored further as positive G4 ligands.

## 4. Conclusions

In the current study, four multi-tasking, multitarget, classification-based QSAR models were developed to predict four essential G4-oriented properties of ligands: G4 selectivity, G4 interaction, G4 stabilization, and cytotoxicity. Their structural features were integrated with diverse experimental conditions by means of the Box–Jenkins moving average approach; the prediction of the activity of a ligand against multiple G4 targets was also made via a single model. The LDA and RF approaches of machine learning were employed to derive the four mtQSAR models. Based on the internal and external validation matrices, the models are found to be robust and have substantial discriminatory power. A user-friendly web platform, ‘G4- QuadScreen’ (as a part of ChemoPredictionsuite platform (https://chemopredictionsuite.com/, accessed 1 June 2023), was developed to screen libraries of compounds against all four mtQSAR models. This tool calculates structural descriptors and predicts G4 selectivity, G4 interaction, G4 stabilization, and cytotoxicity in one single operation and at a fast pace. These four properties are key in defining the G4-mediated anticancer therapeutic effect of any ligand; therefore, the G4-QuadScreen platform offers an easy solution for finding lead molecules against multiple G4s.

Based on the predictions of G4-QuadScreen and results of molecular docking, 62 natural compounds were found to be active against multiple G4s (among *hTel*, *cMYC*, *cKIT1*, *cKIT2*, and *KRAS*). The stabilization capacity and selectivity of the 62 screened compounds against *hTel*, *cMYC*, and *cKIT2* were further evaluated using biophysical assays. Twenty-six out of the sixty-two screened compounds showed a selective stabilization of *cMYC-G4*, and sixteen compounds showed a selective stabilization of *hTel-G4* (selectivity towards G4 over duplex DNA)*. Ten* compounds (Figure S7) showed stabilization against *hTel*, *cMYC*, and *cKIT2 G4s.* However, it should be noted that 10 ligands did not show activity against all 3 tested G4s; instead, 8 out of 10 ligands stabilized *cMYC-G4*, 4 ligands stabilized *hTel-G4*, and 5 ligands stabilized *cKIT2*. The molecular docking and FID results suggest that 10 ligands were bound at multiple binding sites over the respective G4s, such as groove regions and end regions. Furthermore, a cell-based analysis of these 10 ligands suggested that Lig-5, Lig-46, and Lig-48 were active against multiple cancer cell lines. Thus, based on our in silico and in vitro findings, Lig-5, Lig-46, and Lig-48 can be considered lead molecules and must be further explored as potential cancer therapeutic agents.

## Figures and Tables

**Table 1:** Multiple targets crucial in cancer therapy.

Gene	Function	Cancer Type
*cMYC*	Cell proliferation, differentiation, and apoptosis (PDA)	Cervical carcinoma, myeloid leukemia (MyL)
*cKIT*	Cell PDA, motility, adhesion, angiogenesis	Gastrointestinal cancer, MyL
*KRAS*	Cell PDA	Lung, pancreatic cancer, MyL
*BCL2*	Oncogenesis through cell death resistance	Small-cell lung cancer, breast cancer
*hTel*	Cell cycle arrest and cell death	Numerous types of cancer

**Table 2:** Brief details of experimental assays considered in each evaluating model.

Model	Experiment	Outcome
G4 interaction	UV, SPR, fluorescence titrations	The absorption and fluorescence response of the molecules upon binding with G4s and duplex structures allow us to determine binding affinity constants, thus providing information about interaction capacity and the selectivity of ligands towards G4.
G4 stabilization	UV, FRET, CD melting data	∆T_m_ (change in melting temperature) quantitatively determines the stabilization effect of ligands over the secondary structure of DNA. Thus, the G4 stabilization effect and G4 selectivity can be evaluated.
G4 selectivity	Combination of above experiments	When the interaction capacity and stabilization effect are one order higher in G4 than that for duplex DNA, the ligand is considered selective.
Cytotoxicity	MTT, CCK8, MTS	These assays provide *IC_50_* values of ligands against various cancer cell lines, thus providing information on the activity of ligands at a cellular level.

**Table 3:** Pre-defined threshold values for classifying the data into active and inactive categories for each evaluating model.

Model	Threshold
G4 selectivity	When ΔT_m_ and K_d_ values are higher in G4 than those in duplex DNA, the ligand is considered selective, or else non-selective.
G4 interaction	Positive: K_d_ < 1 µM Negative: K_d_ ≥ 1 µM
G4 stabilization	Positive: ΔT_m_ ≥ 15 °C Negative ΔT_m_ < 15 °C
Cytotoxicity	Positive: IC_50_ < 10 µM Negative: IC_50_ ≥ 10 µM

**Table 4:** Internal and external validation parameters of the G4 selectivity model.

Model Category	Parameters	Train	Train 10-Fold CV	Test	Ext	Ext in-AD
I. G4 Selectivity Descriptor: 15 Random forest	No. of Compounds	P: 493 N: 175 Total: 668	P: 493 N: 175 Total: 668	P: 127 N: 39 Total: 166	P: 159 N: 58 Total: 217	P: 156 N: 50 Total: 206
	Accuracy%	99.850	81.280 ± 5.250	86.747	90.320	91.260
	Precision%	99.800	83.860 ± 4.600	90.700	92.590	92.590
	Sensitivity%	100	92.890 ± 4.600	92.130	94.340	96.150
	Specificity%	99.430	48.370 ± 17.250	69.230	79.310	76.000
	F-measure	0.9999	0.880 ± 0.035	0.914	0.935	0.943
	MCC	0.996	0.485 ± 0.158	0.625	0.749	0.755

P = number of datapoints with positive class, N = number of datapoints with negative class, Train = training set, CV = cross validation, Test = test set, Ext = external set, in-AD = inside applicability domain.

**Table 5:** Internal and external validation parameters of the G4 interaction model.

Model Category	Parameters	Train	Train 10-Fold CV	Test	Ext	Ext in-AD
II. G4 Interaction (K_d_) Descriptor: 10 LDA	No. of Compounds	P: 100 N: 95 Total: 195	P: 100 N: 95 Total: 195	P: 21 N: 27 Total: 48	P: 29 N: 25 Total: 54	P: 23 N: 16 Total: 39
	Accuracy%	92.821	86.340 ± 9.734	85.417	83.330	89.740
	Precision%	93.000	84.014 ± 11.390	75.000	88.460	95.240
	Sensitivity%	93.000	91.999 ± 7.483	100.000	79.310	86.960
	Specificity%	92.632	80.670 ± 14.942	74.074	88.000	93.750
	F-measure	0.930	0.875 ± 8.660	0.857	0.836	0.909
	MCC	0.856	0.736 ± 18.770	0.745	0.672	0.796

P = number of datapoints with positive class, N = number of datapoints with negative class, Train = training set, CV = cross validation, Test = test set, Ext = external set, in-AD = inside applicability domain.

**Table 6:** Internal and external validation parameters of the G4 stabilization model.

Model Category	Parameters	Train	Train 10-Fold CV	Test	Ext	Ext in-AD
III. G4 Stabilization (ΔT_m_) Threshold Positive: ΔT_m_ ≥ 15 °C Negative: ΔT_m_ < 15 °C Descriptor: 10 Random Forest	No. of Compounds	P: 498 N: 394 Total: 892	P: 498 N: 394 Total: 892	P: 129 N: 93 Total: 222	P: 154 N: 121 Total: 275	P: 119 N: 85 Total: 204
	Accuracy%	99.890	85.540 ± 4.970	90.540	85.091	85.440
	Precision%	100.000	86.990 ± 7.170	92.860	88.970	88.650
	Sensitivity%	99.800	88.335 ± 7.790	90.700	83.770	85.030
	Specificity%	100.000	81.885 ± 11.860	90.320	86.780	85.960
	F-measure	0.999	0.872 ± 0.044	0.918	0.863	0.868
	MCC	0.998	0.714 ± 0.098	0.807	0.701	0.707

P = number of datapoints with positive class, N = number of datapoints with negative class, Train = training set, CV = cross validation, Test = test set, Ext = external set, in-AD = inside applicability domain.

**Table 7:** Internal and external validation parameters of the cytotoxicity model.

Model Category	Parameters	Train	Train 10-Fold CV	Test	Ext	Ext in-AD
IV. Cytotoxicity (MTT, MTS, CCK8) Threshold Positive: IC_50_ < 10 µM Negative: IC_50_ ≥ 10 µM Descriptor: 9 Random Forest	No. of Compounds	P: 244 N: 219 Total: 463	P: 244 N: 219 Total: 463	P: 71 N: 44 Total: 115	P: 79 N: 65 Total: 144	P: 74 N: 65 Total: 139
	Accuracy%	100.000	69.740 ± 6.520	84.348	83.330	82.730
	Precision%	100.000	74.285 ± 12.010	92.060	85.710	83.560
	Sensitivity%	100.000	71.000 ± 18.620	81.690	83.540	83.560
	Specificity%	100.000	68.440 ± 20.270	88.640	83.080	81.820
	F-measure	1.000	0.870 ± 0.088	0.866	0.846	0.836
	MCC	1.000	0.426 ± 0.144	0.687	0.665	0.654

P = number of datapoints with positive class, N = number of datapoints with negative class, Train = training set, CV = cross validation, Test = test set, Ext = external set, in-AD = inside applicability domain.

**Table 8:** Screening criteria of specific G4 targets, experimental conditions, etc., for each evaluating model.

G4 Model	Screening Criteria	Rationale
Selectivity	**G4 Sequences:** *hTel*: AGGGTTAGGGTTAGGGTTAGGG *hTel:* GGGTTAGGGTTAGGGTTAGGG *cMYC:* TGAGGGTGGGTAGGGTGGGTAA *cKIT1*: GGGAGGGCGCTGGGAGGAGGG *cKIT2*: GGGCGGGCGCGAGGGAGGGG *KRAS*: AGGGCGGTGTGGGAAGAGGGAAGAGGGGGAGG	~50% of the modeling data comprise these sequences.
	**Buffer:** KCl 10 mM LiCl 90 mM Lithium Cacodylate (10 mM) NaCl 100 mM Lithium Cacodylate (10 mM) KCl 100 mM Lithium Cacodylate (10 mM)	Frequently adopted in FRET experiments, and cover parallel, antiparallel, and hybrid topology of G4.
	**Assay:** FRET melting	~43% of the modeling data are derived from FRET experiments.
Interaction	**G4 Sequences:** *hTel*: AGGGTTAGGGTTAGGGTTAGGG *hTel:* GGGTTAGGGTTAGGGTTAGGG *cMYC:* TGAGGGTGGGTAGGGTGGGTAA *cKIT1*: GGGAGGGCGCTGGGAGGAGGG *cKIT2*: GGGCGGGCGCGAGGGAGGGG *KRAS*: AGGGCGGTGTGGGAAGAGGGAAGAGGGGGAGG	~50% of the modeling data comprise these sequences.
	**Buffer:** KCl 100 mM Lithium Cacodylate (10 mM) NaCl 35 mM KCl 50 mM Tween20 0.05% HEPES (10 mM) NaCl 100 mM Tris-HCl (50 mM) KCl 100 mM Tris-HCl (10 mM)	Frequently adopted in G4-*K_d_* studies, and cover parallel, antiparallel, and hybrid topology of G4.
Stabilization	**G4 Sequences:** *hTel*: AGGGTTAGGGTTAGGGTTAGGG *hTel:* GGGTTAGGGTTAGGGTTAGGG *cMYC:* TGAGGGTGGGTAGGGTGGGTAA *cKIT1*: GGGAGGGCGCTGGGAGGAGGG *cKIT2*: GGGCGGGCGCGAGGGAGGGG *KRAS*: AGGGCGGTGTGGGAAGAGGGAAGAGGGGGAGG	~55% of the modeling data comprise these sequences.
	**Buffer:** KCl 10 mM LiCl 90 mM Lithium Cacodylate (10 mM) NaCl 10 mM LiCl 90 mM Lithium Cacodylate (10 mM)	Frequently adopted in FRET experiments, and cover parallel, antiparallel, and hybrid topology of G4.
	**Assay:** FRET melting experiments	~88% of the modeling data comprise this assay condition.
	**Ligand to G4 ratio (LGR):** 5, 10	~53% of the modeling data were obtained for these LGRs, and they have well-balanced class distribution.
Cytotoxicity	**Cell lines:** *HELA, A549, MCF7, A375, HCT116*	~48% of the modeling data have the cell line condition of these cell lines.
	**Exposure time:** 48, 72 h	~95% of the modeling data have these exposure time conditions.
	**Assay:** MTT	~90% of the modeling data are derived from the MTT assay.

**Table 9:** Summary of molecular docking results; average binding energies of 62 ligands in their respective G4 receptors and their respective binding pockets.

G4 Motif	Average Binding Energies (kcal/mol)	G4 Pocket
*hTel _(antiparallel)_*	9.11	5′end, Groove
*hTel _(3+1 hybrid)_*	7.63	Groove
*hTel _(parallel)_*	7.63	3′end, Groove
*cMYC*	7.83	5′end, 3′end, Groove
*cKIT1*	8.41	3′end, Groove
*cKIT2*	7.46	5′end, 3′end, Groove
*KRAS*	7.77	5′end, 3′end, Groove

**Table 10:** IC_50_ values obtained from the lead compounds in the indicated cell lines treated for 48 (^a^) or 72 h (^b^).

Compounds	IC_50_ (µM)				
	A549 ^a^	MCF-7 ^a^	HeLa ^a^	GIST T1 ^b^	GIST 430/650 ^b^
**5**	>100	11.8	>100	16.01	22.94
**46**	12.24	6.02	15.17	25.11	8.495
**48**	51.58	25.95	62.64	15.74	26.65

## Data Availability

The data generated in this research are available at https://osf.io/tgchf/.

## Supplementary Materials

The following supporting information can be downloaded at: https://www.mdpi.com/article/10.3390/cancers15153817/s1. Figure S1. Structural features of G4: (A) Hoogsteen hydrogen-bonding among guanine bases form a planar arrangement, and partial negative charges become accumulated towards the central core of the tetrad due to carbonyl oxygen atoms. (B) Guanine planes stack over each other and become intercalated via counterbalancing metal ions. (C) Few among various topologies of G4, based on the orientation of guanine strands (green arrows) and arrangement of interconnecting loops (blue dotted line), have variations in topologies. Figure S2. (A) Schematic representation of significance of G4 at the 3′ telomeric end in cancer therapy. (B) Schematic representation of significance of G4 at the promoter region of oncogenes in cancer therapy. Figure S3. The binding energy of each ligand against various DNA motifs was estimated using molecular docking. Each graph has binding energies of a set of molecules as labeled over a respective graph. Color codes for each DNA motif are enlisted at the right bottom. Figure S4. Representation of FRET melting values (ΔT_m_) for the interaction between the ligands and ds26. The concentration of DNA was 0.2 μM, and the [DNA]/[Ligand] ratios were 1:10 and 1:20. Errors denote the standard deviations of at least three independent experiments. Figure S5. Plots of dose–response curves of the ligands for A549 (top panel), MCF-7 (middle panel), and HeLa (bottom panel) cancer cell lines. The mean ± SD values from three independent experiments, each conducted in triplicate, are shown in the graph, representing the percentage of viable cells. Figure S6. Plots of dose–response curves of the ligands for GIST T1 (top panel) and GIST 430/650 (bottom panel) cancer cell lines. Data are expressed as mean ± SD (*n* = 3 independent assays). The mean ± SD values from three independent experiments, each conducted in triplicate, are shown in the graph, representing the percentage of viable cells. Figure S7. Chemical structures of the hit ligands. Table S1. Parameters of each evaluating model. Table S2. PDBs utilized in molecular docking. Table S3. Information of each descriptor contributing to the G4 selectivity model. Table S4. Information of each descriptor contributing to the G4-interaction model. Table S5. Information of each descriptor contributing to the G4-stabilization model. Table S6. Information of each descriptor contributing to the cytotoxicity model. Table S7. Sequences of the nucleic acids, topology and genome localization. Table S8. Percentages (%) of TO displaced upon addition of the ligands, concentration for each ligand is as indicated in the bracket (in µM). Table S9. Binding sites of hTel and cMYC where selected ligands are interacting in their best-docked pose. Supplimentary_Docking-results.xlsx. Details of the binding energies of each ligand and binding residue with respective DNA motifs.
