# Supplementary material for: G4-QuadScreen: A Computational Tool for Identifying Multi-Target-Directed Anticancer Leads against G-Quadruplex DNA
Source: Cancers (Basel). 2023 Jul 27;15(15):3817. doi: 10.3390/cancers15153817 (PMC10416877; doi:10.3390/cancers15153817)
Supplement: Supplementary file 1 [file cancers-15-03817-s001.zip › Graphical-abstract.pptx]

## Slide 1
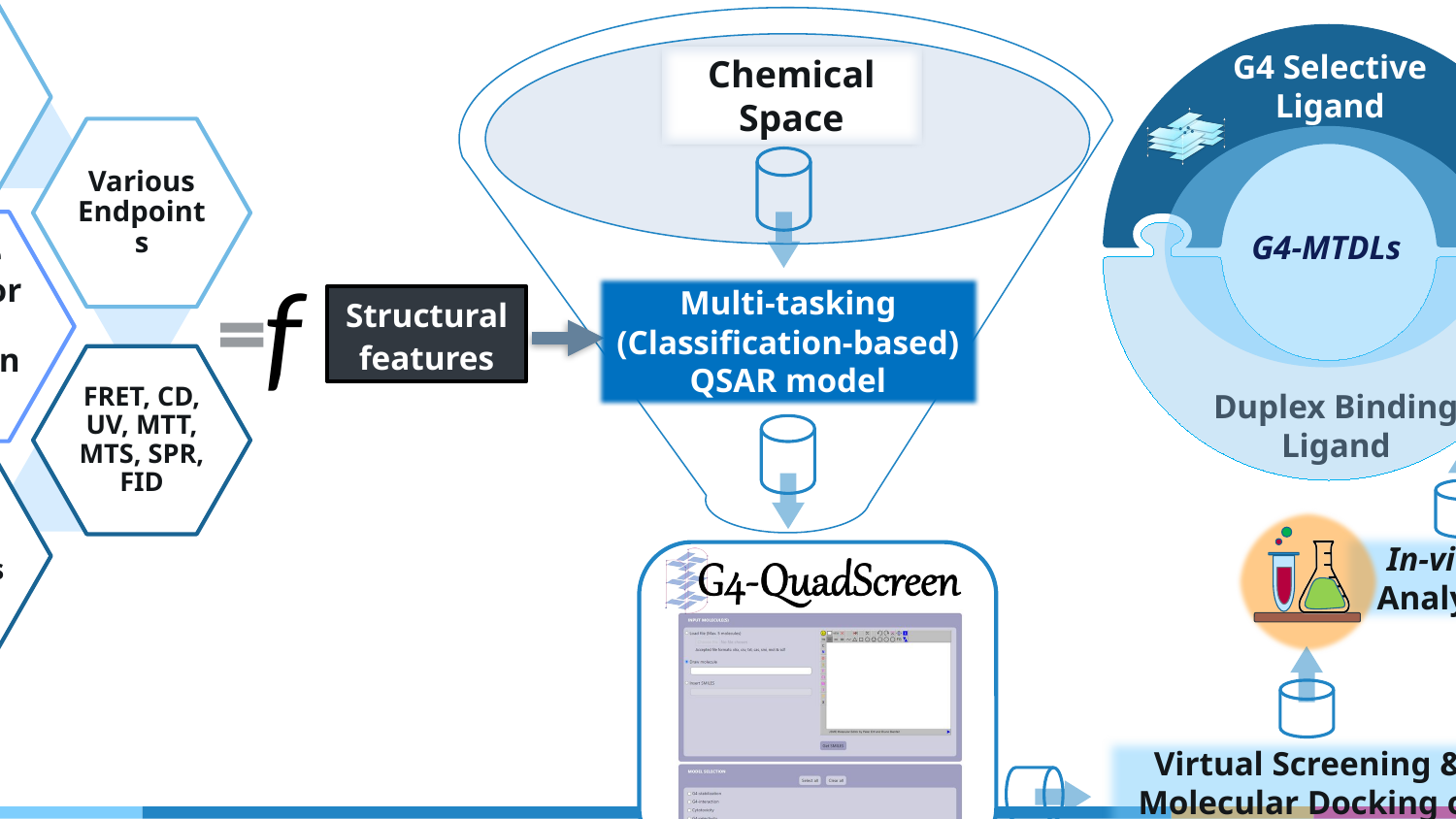

G4 Selective
Ligand
Duplex Binding Ligand
Chemical Space
Multi-tasking
(Classification-based)
 QSAR model
G4-MTDLs
f
Structural features
In-vitro Analysis
Virtual Screening &
Molecular Docking of
MTDL
