# Supplementary material for: G4-QuadScreen: A Computational Tool for Identifying Multi-Target-Directed Anticancer Leads against G-Quadruplex DNA
Source: Cancers (Basel). 2023 Jul 27;15(15):3817. doi: 10.3390/cancers15153817 (PMC10416877; doi:10.3390/cancers15153817)
Supplement: Supplementary file 1 [file cancers-15-03817-s001.zip › Supplimentary/Supplimentary-material JGG.pdf]

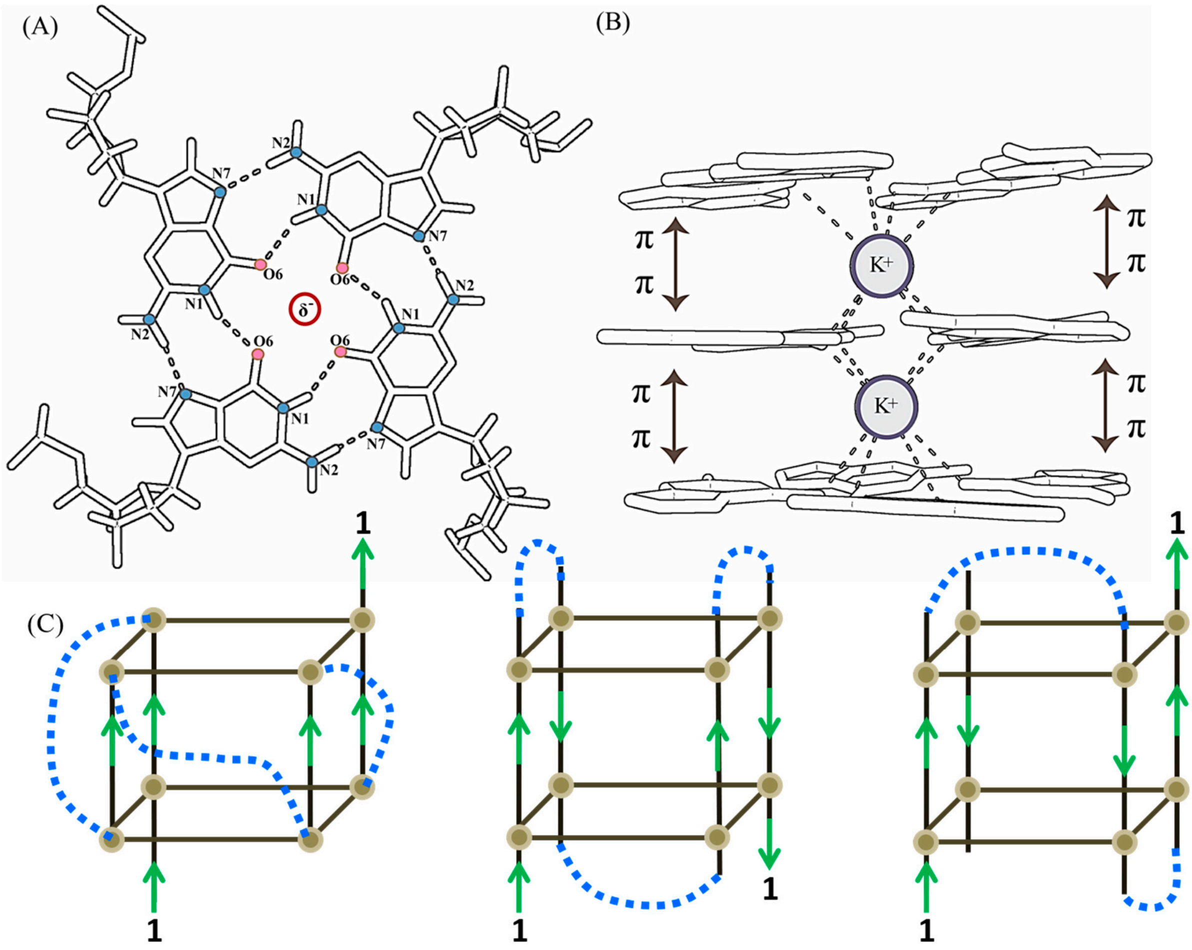

Figure-S1. Structural features of G4:(A) Hoogsteen hydrogen bonding among guanine bases form planar arrangement, partial negative charges get accumulated towards the central core of the tetrad due to carbonyl oxygen atoms, (B) guanine planes stack over each other and get intercalated by counterbalancing metal ions, (C) few among various topologies of G4, based on the orientation of guanine strands (green arrows) and arrangement of interconnecting loops (blue dotted line) G4s attain variation in topologies

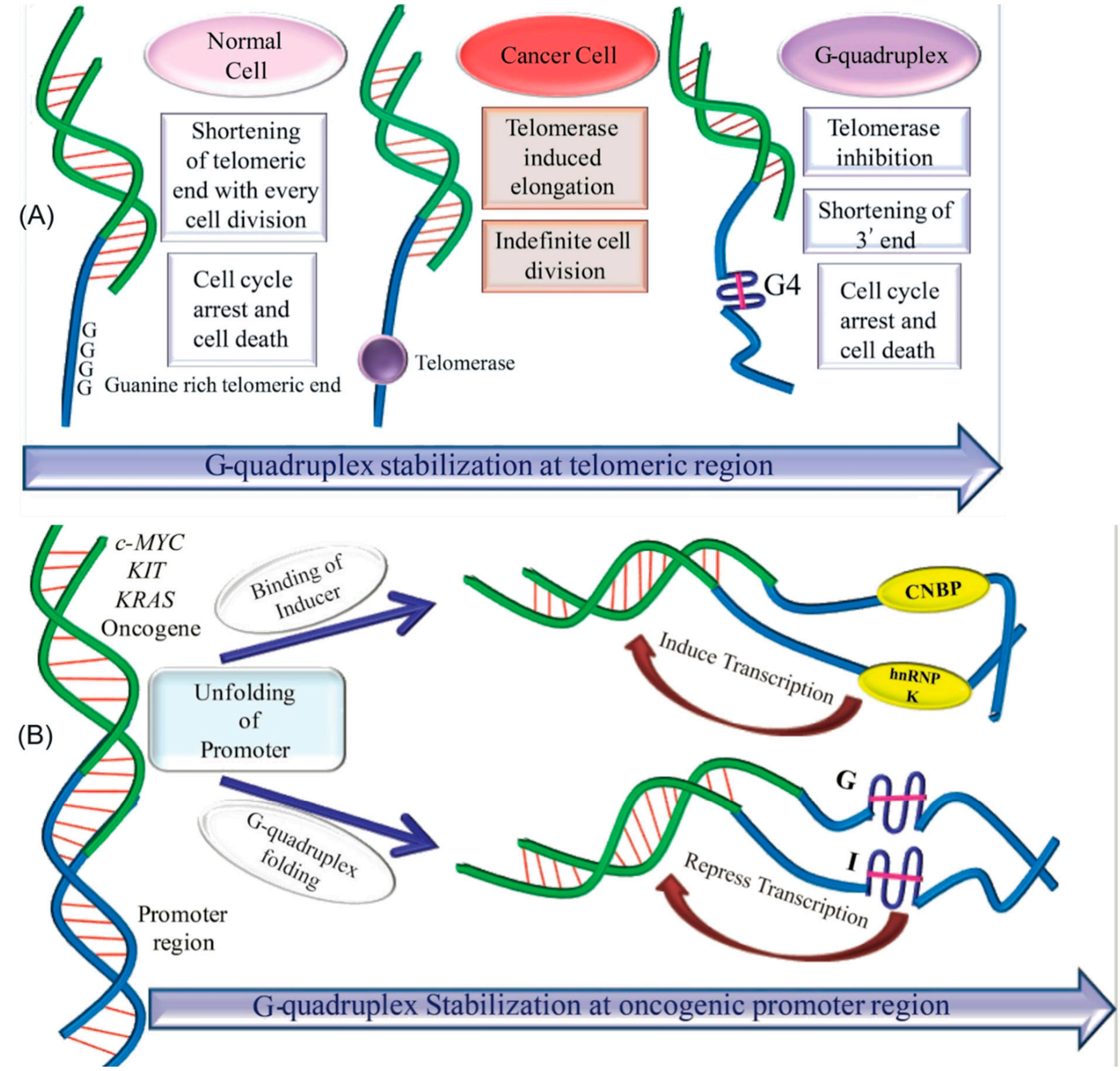

Figure S2 (A) Schematic representation of significance of G4 at the 3' telomeric end in cancer therapy, (B) schematic representation of significance of G4 at the promoter region of oncogenes in cancer therapy

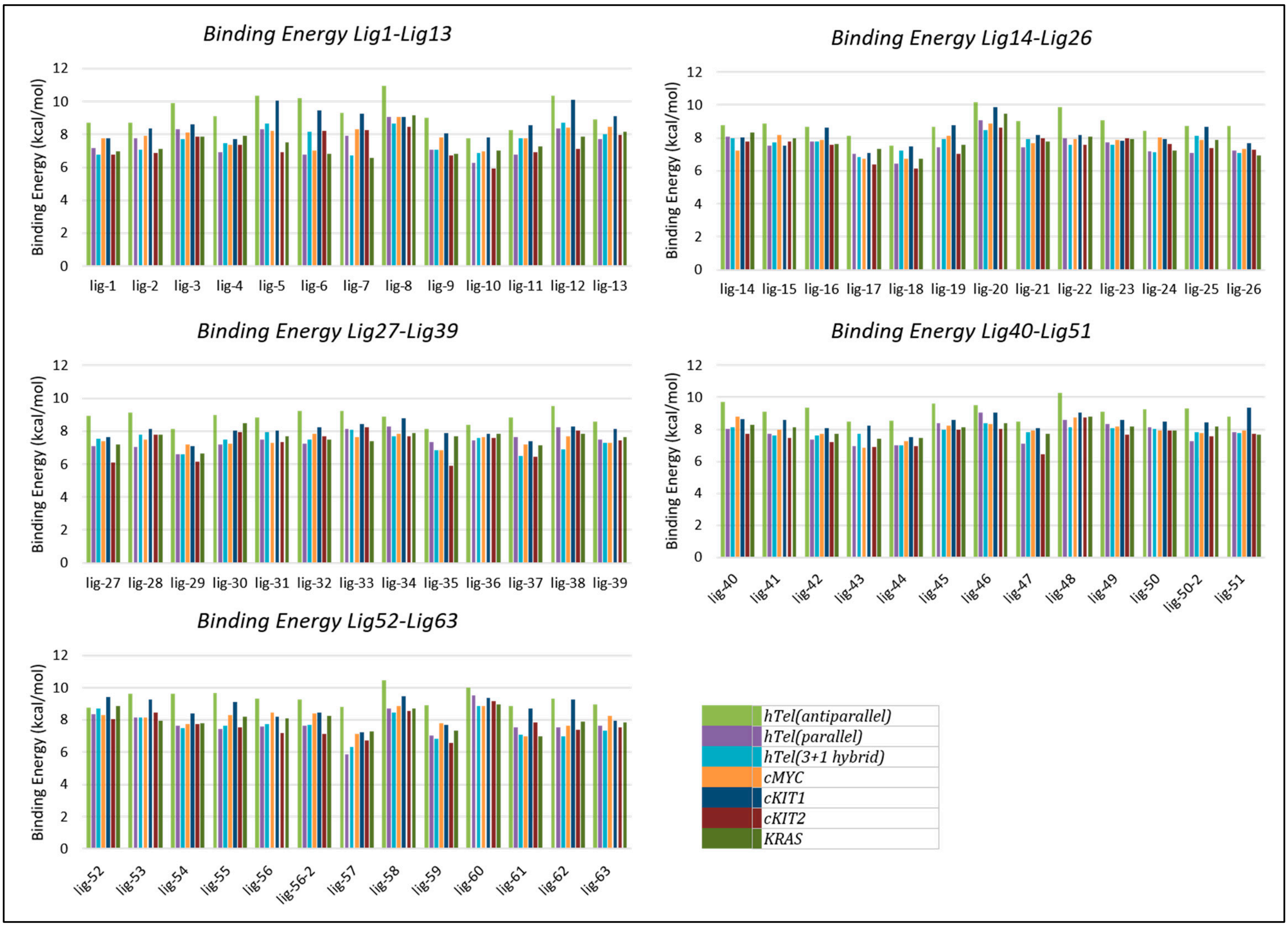

Figure-S3. The binding energy of each ligand against various DNA motifs was estimated using molecular docking. Each graph has binding energies of a set of molecules as labeled over a respective graph. Color codes for each DNA motif are enlisted at the right bottom

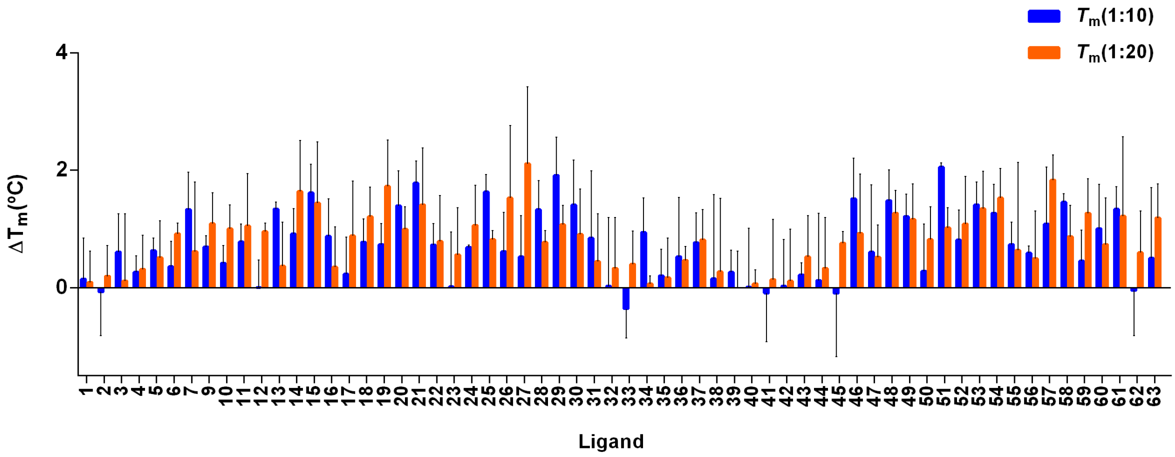

Figure-S4. Representation of FRET melting values (*ΔTm*) for the interaction between the ligands and *ds26*. The concentration of DNA was 0.2 μM and the [DNA]:[Ligand] ratios were 1:10 and 1:20. Errors denote the standard deviations of at least three independent experiments.

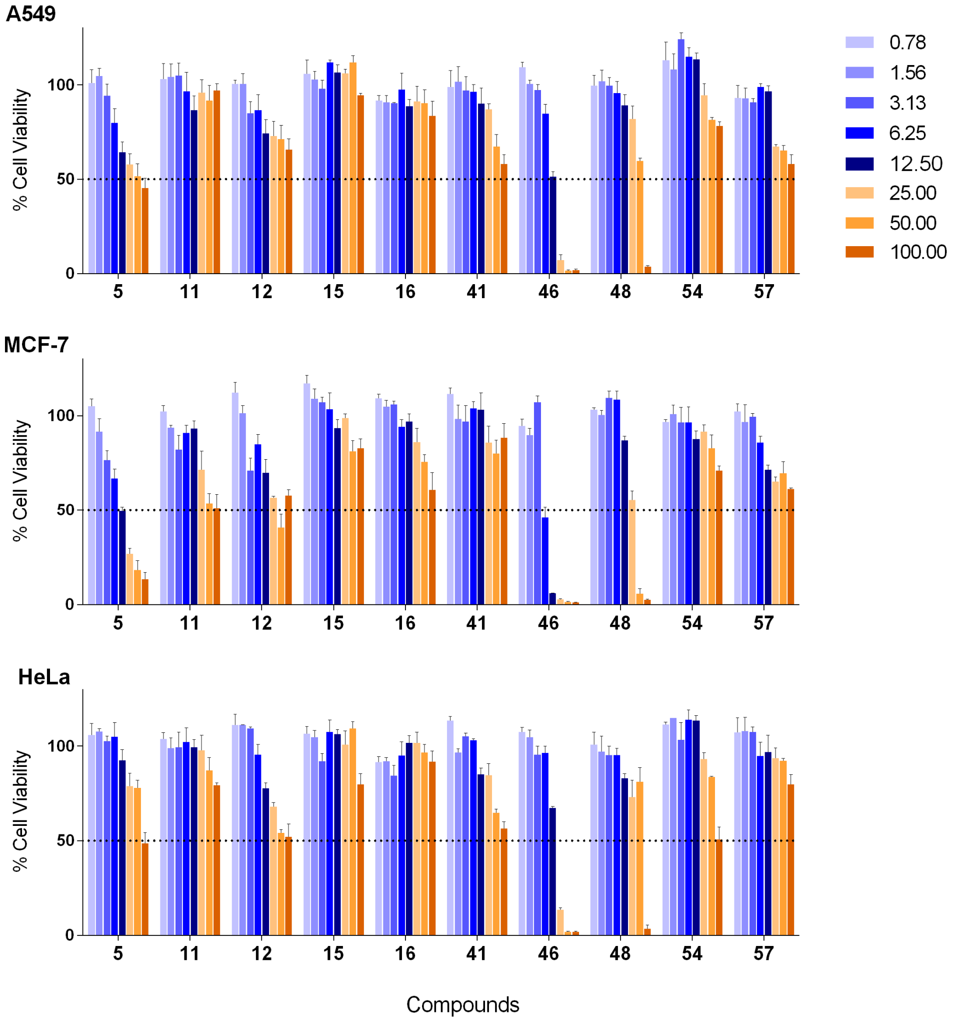

Figure-S5. Plots of dose-response of the ligands for A549 (top panel), MCF-7 (middle panel), HeLa (bottom panel) cancer cell lines. The mean ± SD values from three independent experiments, each conducted in triplicate, are shown in the graph, representing the percentage of viable cells

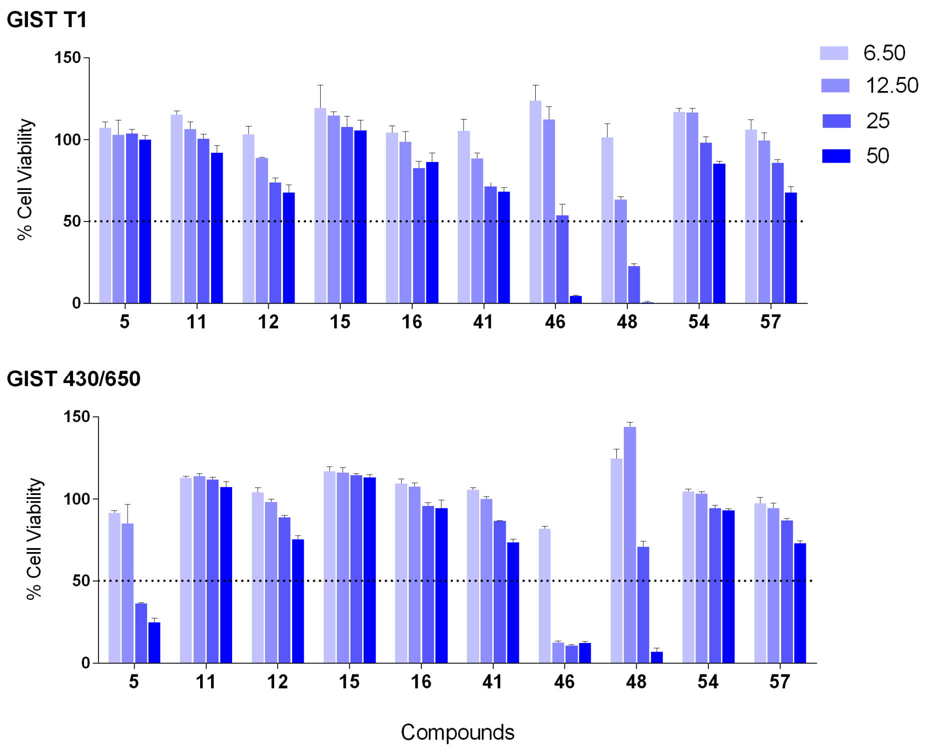

Figure-S6. Plots of dose-response of the ligands for GIST T1 (top panel) and GIST 430/650 (bottom panel) cancer cell lines. Data are expressed as mean ± SD (n=3 independent assays). The mean ± SD values from three independent experiments, each conducted in triplicate, are shown in the graph, representing the percentage of viable cells.

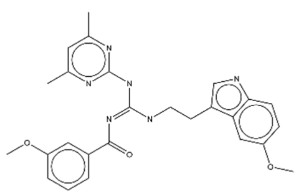

Lig-5

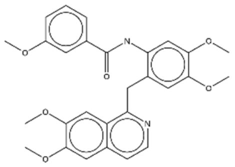

Lig-11

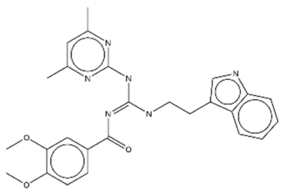

Lig-12

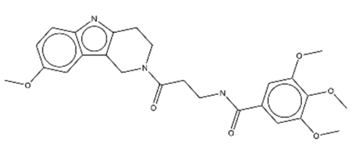

Lig-15

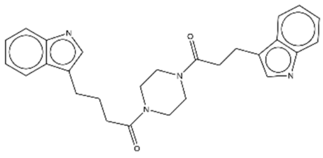

Lig-16

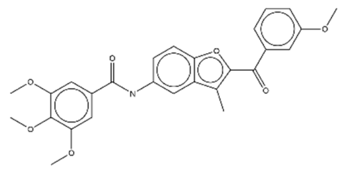

Lig-41

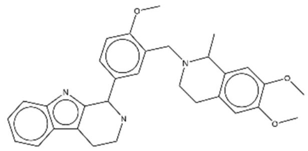

Lig-46

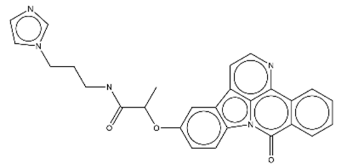

Lig-48

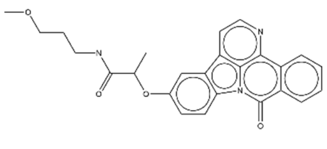

Lig-54

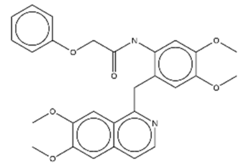

Lig-57

Figure-S7. Chemical structures of the *hit* ligands.

Table-S1. Parameters of each evaluating model

Model	Modeling Parameters
G4-selectivity	Random Forest (random state=0, max depth=20, n_estimators=50)
G4-interaction	Linear discriminant analysis (default parameters)
G4-stabilization	Random Forest (random state=1)
Cytotoxicity	Random Forest (random state=0, max depth=20, n_estimators=50)

Table-S2. PDBs utilized in molecular docking

G4-oncogene	PDB ID	Topology
hTel	2MCO	Anti-parallel
hTel	2JSM	Hybrid-3+1
hTel	4DA3	Parallel
cMYC	1XAV	Parallel
cKIT1	2O3M	Parallel
cKIT2	2KQH	Parallel
KRAS	6SUU	Parallel

Table-S3. Information of each descriptor contributing to the G4-selectivity model

Descriptor name	Source	Corresponding condition	Feature importance	Meaning	Type
SpMAD_v	in-house	Gene sequence	0.135±0.054	Spectral mean absolute deviation from Barysz matrix weighted by vdW volume	Barysz matrix 2D
R1p	in-house	Gene sequence	0.102±0.036	R autocorrelation of lag 1 / weighted by polarizability	GETAWAY
BELd-1	in-house	Buffer	0.095±0.034	Lowest eigenvalue of Burden matrix weighted by sigma electronics	Burden Eigenvalues
GATS8i	in-house	Oncogene	0.088±0.028	Geary autocorrelation of lag 8 (log function) weighted by ionization potential	Autocorrelation 2D
F06[N-O]	in-house	Gene sequence	0.078±0.041	Frequency of N-O at topological distance 6	Two dimensional
SubFP180	PaDEL	Buffer	0.070±0.035	Hetero N basic no H, presence of SMART pattern [nX3H0+0]	Substructure Fingerprinter
F04[N-O]	in-house	Assay	0.068±0.038	Frequency of N-O at topological distance 4	Two Dimensional
PubchemFP260	PaDEL	Gene sequence	0.063±0.034	>= 3 Hetero-aromatic rings	Pubchem Fingerprint
PEOE_VSA12	in-house	Assay	0.060±0.035	MOE charge VSA descriptor 12	MoeType
PubchemFP21	PaDEL	Gene sequence	0.057±0.033	>= 8 O	Pubchem Fingerprint
PubchemFP386	PaDEL	Assay	0.053±0.033	Presence/absence of 'C-O'	Pubchem Fingerprint
KRFP3763	PaDEL	Oncogene	0.049±0.028	Presence/absence of 'CCN(CC)CCN'	Klekota Roth Fingerprinter
B09[O-O]	in-house	Buffer	0.036±0.022	Presence/absence of O-O at topological distance 9	Two dimensional
KRFP3408	PaDEL	Assay	0.032±0.020	Presence/absence of 'CC(C)O'	Klekota Roth Fingerprinter
KRFP3708	PaDEL	Assay	0.015±0.015	Presence/absence of 'CCCCC(=O)O'	Klekota Roth Fingerprinter

Table-S4. Information of each descriptor contributing to the G4-interaction model

Descriptor Name	Source	Corresponding Condition	LDA Coefficient	Meaning	Type
KRFPC3529	PaDEL	Buffer	0.759447527	Count of 'Cc1ccc(C)cc1'	Klekota Roth Fingerprinter
SIC2	*in-house*	Buffer	-4.1781084	Structural information content index (neighborhood symmetry of 2-order)	Information Indices
KRFPC1642	PaDEL	Buffer	-0.71630366	Count of '[!#1]c1[cH][cH]c([!#1])[cH][cH]1'	Klekota Roth Fingerprinter
PubchemFP663	PaDEL	Gene Sequence	-3.112947971	Presence/absence of 'O-C-C-O-[#1]'	Pubchem Fingerprint
R3u	*in-house*	Gene Sequence	-0.883890498	R autocorrelation of lag 3 / unweighted	GETAWAY
piPC10	*in-house*	Oncogene	0.434070494	molecular multiple path count of order 10	Walk Path Counts
TDB08e	*in-house*	Oncogene	-1.556963919	3D Topological distance-based descriptor of lag 08, Sanderson electronegativity-weighted	Rdkit3D
VSA_EState1	*in-house*	Buffer	0.047065222	VSA EState descriptor 1	MoeType
KRFPC3712	PaDEL	Gene Sequence	-0.368292843	count of 'CCCCCC'	Klekota Roth Fingerprinter
KRFP3733	PaDEL	Buffer	1.942929416	Presence/absence of 'CCCCN'	Klekota Roth Fingerprinter

Table-S5. Information of each descriptor contributing to the G4-stabilization model

Descriptor Name	Source	Corresponding Condition	Feature importance	Meaning	Type
SubFP296	PaDEL	Ligand to G4 ratio	0.131±0.034	Presence/absence of charge	Substructure Fingerprinter
X5solA	*in-house*	Gene Sequence	0.172±0.036	Average solvation connectivity index of order 5	Connectivity index
KRFPC4269	PaDEL	Assay	0.024±0.018	Count of 'Nc1ccccn1'	Klekota Roth Fingerprinter
X0vA	*in-house*	Ligand to G4 ratio	0.121±0.032	Average valence connectivity index of order 0	Connectivity index
KRFPC383	PaDEL	Buffer	0.125±0.039	Count of '[!#1][CH2][CH2][NH][!#1]'	Klekota Roth Fingerprinter
TDB04u	*in-house*	Buffer	0.235±0.056	3D Topological distance-based descriptor of lag 04, unweighted	Rdkit3D
PubchemFP147	PaDEL	Ligand to G4 ratio	0.018±0.011	>= 1 unsaturated non-aromatic carbon-only ring size 5	Pubchem Fingerprint
KRFPC1934	PaDEL	Oncogene	0.056±0.019	Count of '[!#1]c1[cH]c([!#1])c([!#1])c([!#1])n1'	Klekota Roth Fingerprinter
PubchemFP545	PaDEL	Gene Sequence	0.061±0.021	Presence/absence of 'N-C:C-C'	Pubchem Fingerprint
KRFP3713	PaDEL	Oncogene	0.056±0.015	Presence/absence of 'CCCCCC(=O)N'	Klekota Roth Fingerprinter

Table-S6. Information of each descriptor contributing to the cytotoxicity model

Descriptor Name	Source	Corresponding Condition	Feature importance	Meaning	Type
F03[N-N]	*in-house*	Cell line	0.083±0.035	Frequency of N-N at topological distance 3	Two dimensional
N-066	*in-house*	Cell line	0.065±0.030	Count of 'Al-NH2', *i.e.* NH2 attached to aliphatic chain	Atom centered
MATS5s	*in-house*	Cell line	0.079±0.042	Moran autocorrelation of lag 5 (log function) weighted by I-state, measures spatial autocorrelation based on both feature locations and feature values simultaneously	Autocorrelation
SIC5	*in-house*	Assay	0.096±0.063	Structural information content index (neighborhood symmetry of 5-order)	Information indices
Mor26u	*in-house*	Assay	0.085±0.046	3D MoRSE signal 26, unweighted	Rdkit3D
TDB04p	*in-house*	Assay	0.120±0.075	3D Topological distance-based descriptor of lag 04, polarizability-weighted	Rdkit3D
H0s	*in-house*	Cell line	0.205±0.0939	H autocorrelation of lag 0 /weighted by I-state	GETAWAY
Variation	*in-house*	Assay	0.130±0.081	Variation	Topological
EEig02u	*in-house*	Exposure time	0.137±0.077	eigenvalue of order 2 from the edge adjacency matrix unweighted	Edge Adjacency

Table-S7. Sequences of the nucleic acids, topology and genome localization

Abreviation	*Sequence (5′ → 3′)*	*Topology*	*Localization*
hTelo	GGGTTAGGGTTAGGGTTAGGG	Hibrid or antiparallel	telomere
cMyc	TGAGGGTGGGTAGGGTGGGTAA	Parallel	promoter of cMyc
cKit2	CGGGCGGGCGCGAGGGAGGGG	Parallel	promoter of kit
ds26	CAATCGGATCGAATTCGATCCGATTG	B-type duplex	

Table-S8. Percentages (%) of TO displaced upon addition of the ligands, concentration for each ligand is as indicated in the bracket (in μM)

**Ligands**	*hTel*	*cMYC*
**Lig-5**	19.27(20.59)	**43.15(11.85)**
**Lig-11**	9.20(20.59)	11.97(11.85)
**Lig-12**	0.07(11.85)	12.05(16.30)
**Lig-15**	-38.58(11.38)	6.6(11.85)
**Lig-16**	-19.70(11.85)	-19.66(11.85)
**Lig-41**	-24.70(7.22)	16.48(16.30)
**Lig-46**	**29.53(89.74)**	-4.8(11.85)
**Lig-48**	16.35(16.30)	**28.56(20.59)**
**Lig-54**	2.80(11.85)	-0.17(11.85)
**Lig-57**	-5.13(7.22)	2.51(11.85)

Table-S9. Binding sites of *hTel* and *cMYC* where selected ligands are interacting in their best-docked pose

**Ligands**	***hTel (Hybrid 3+1)***	***hTel (parallel)***	***cMYC***
**Lig-5**	Groove Binding	3'-end, groove	5'-end, groove
**Lig-11**	Groove Binding	3'end	5'-end, groove
**Lig-12**	Groove Binding	3'-end, groove	5'-end, groove
**Lig-15**	Groove Binding	3'-end, groove	3'-end, groove
**Lig-16**	Groove Binding	3'-end, groove	3'-end, groove
**Lig-41**	Groove Binding	3'-end, groove	5'-end
**Lig-46**	Groove Binding	3'-end, groove	5'-end, groove
**Lig-48**	Groove Binding	3'-end, groove	5'-end
**Lig-54**	Groove Binding	3'-end, groove	groove
**Lig-57**	Groove Binding	groove	5'-end, groove
