# Supplementary figures and images for: G4-QuadScreen: A Computational Tool for Identifying Multi-Target-Directed Anticancer Leads against G-Quadruplex DNA
Source: Cancers (Basel). 2023 Jul 27;15(15):3817. doi: 10.3390/cancers15153817 (PMC10416877; doi:10.3390/cancers15153817)

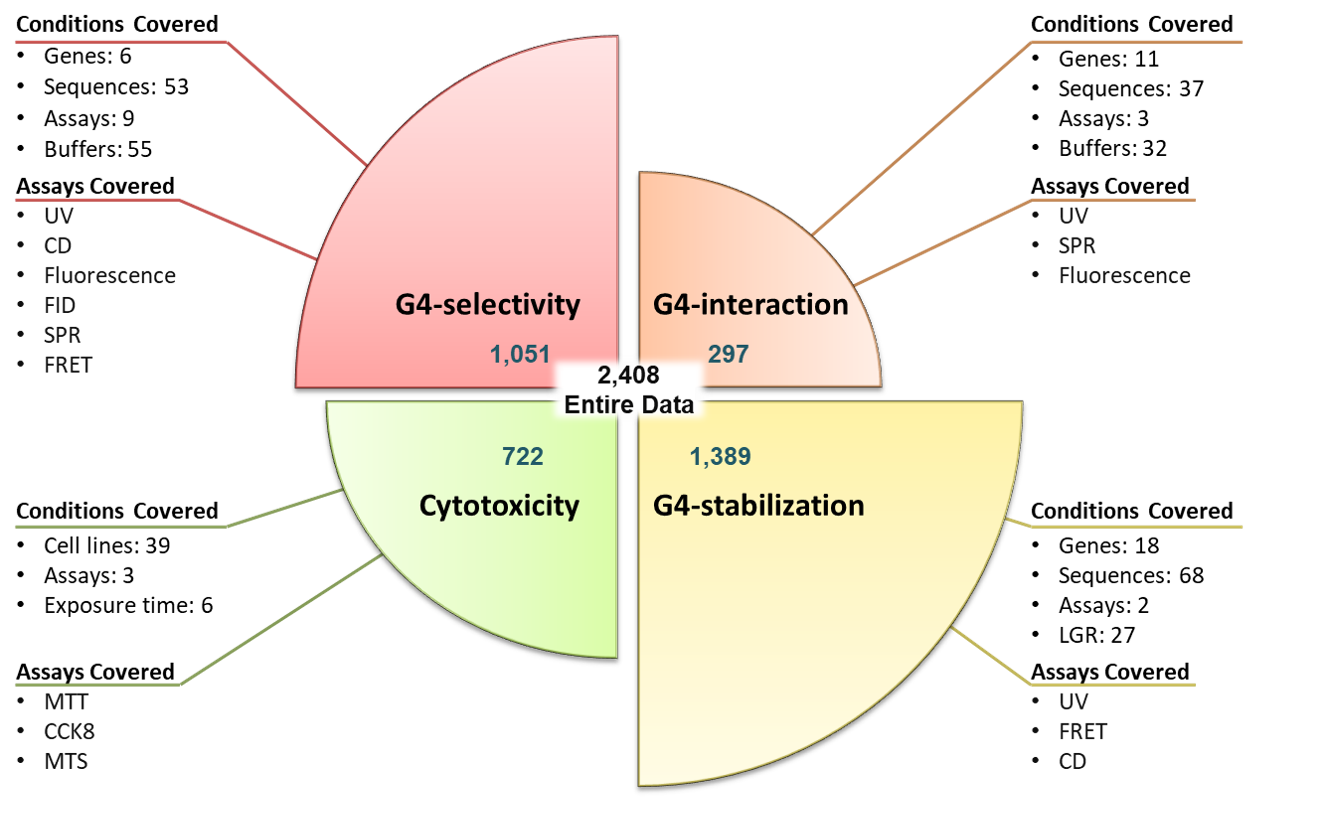

Supplement: Supplementary file 1 [file cancers-15-03817-s001.zip › Fig1.png]

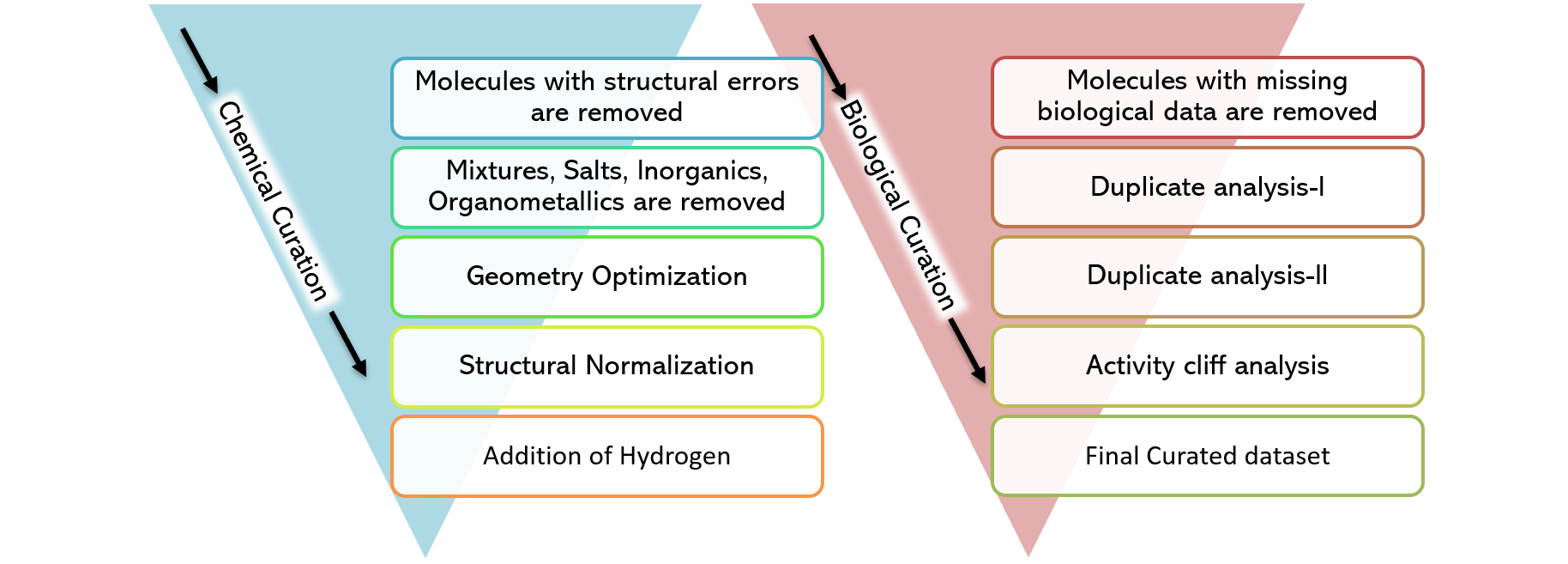

Supplement: Supplementary file 1 [file cancers-15-03817-s001.zip › Fig2.png]

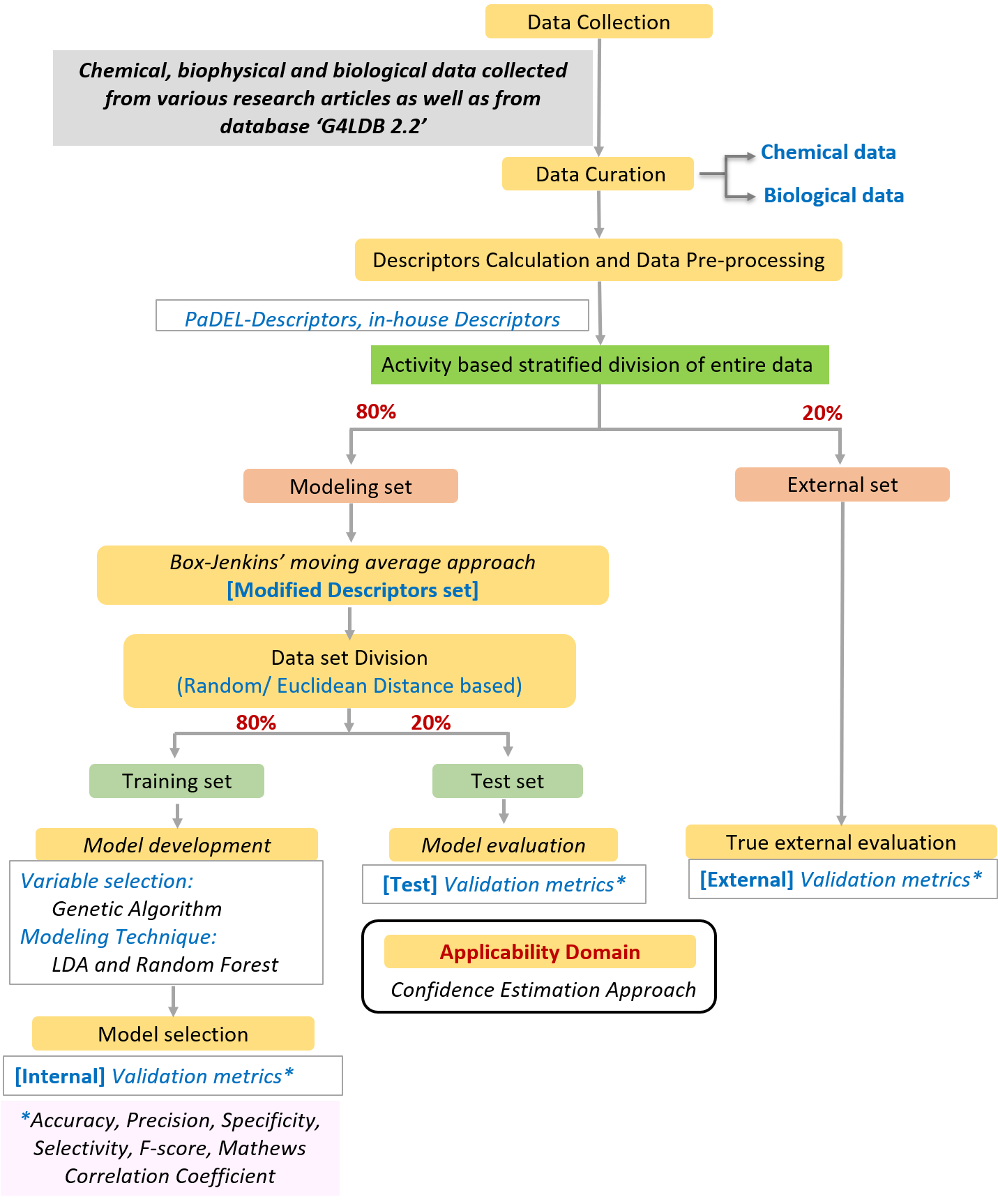

Supplement: Supplementary file 1 [file cancers-15-03817-s001.zip › Fig3.png]

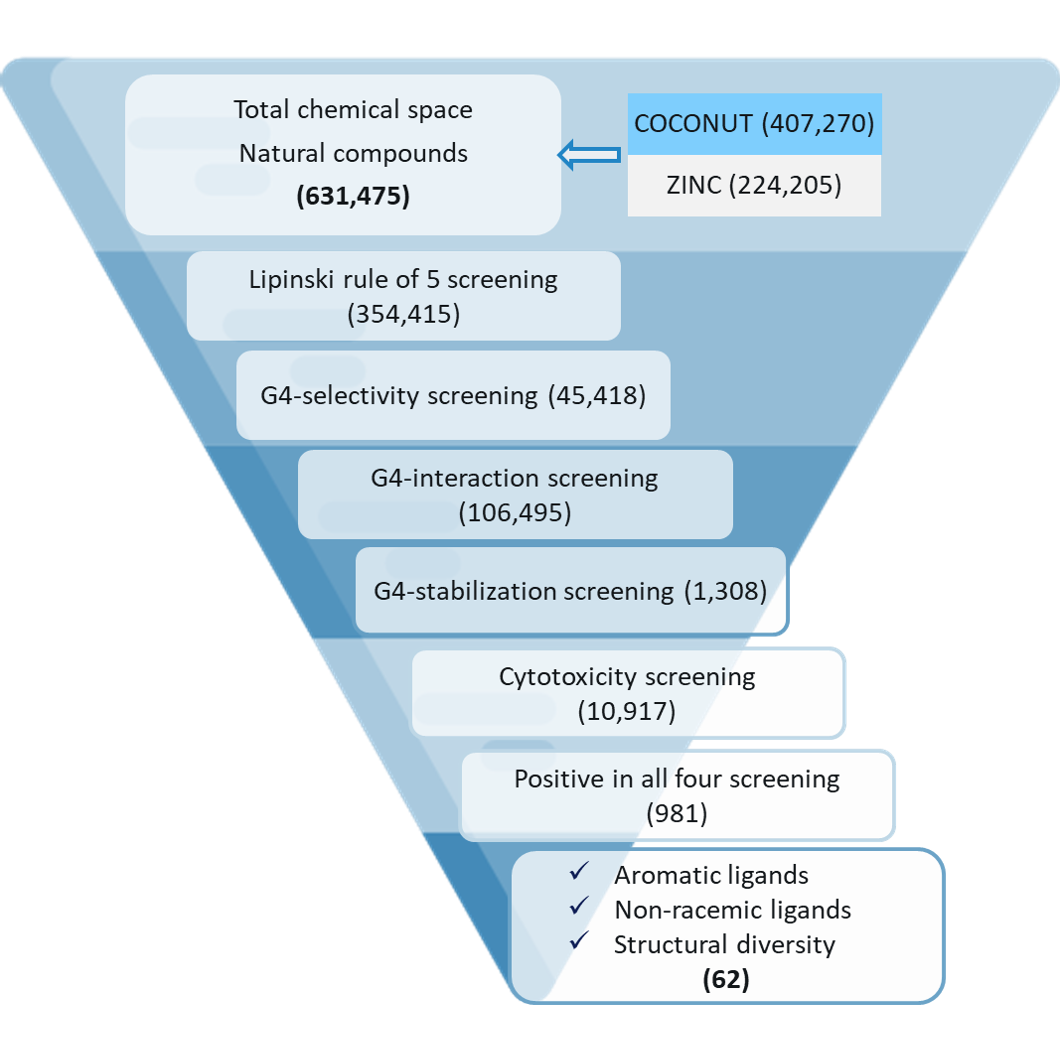

Supplement: Supplementary file 1 [file cancers-15-03817-s001.zip › Fig4.png]

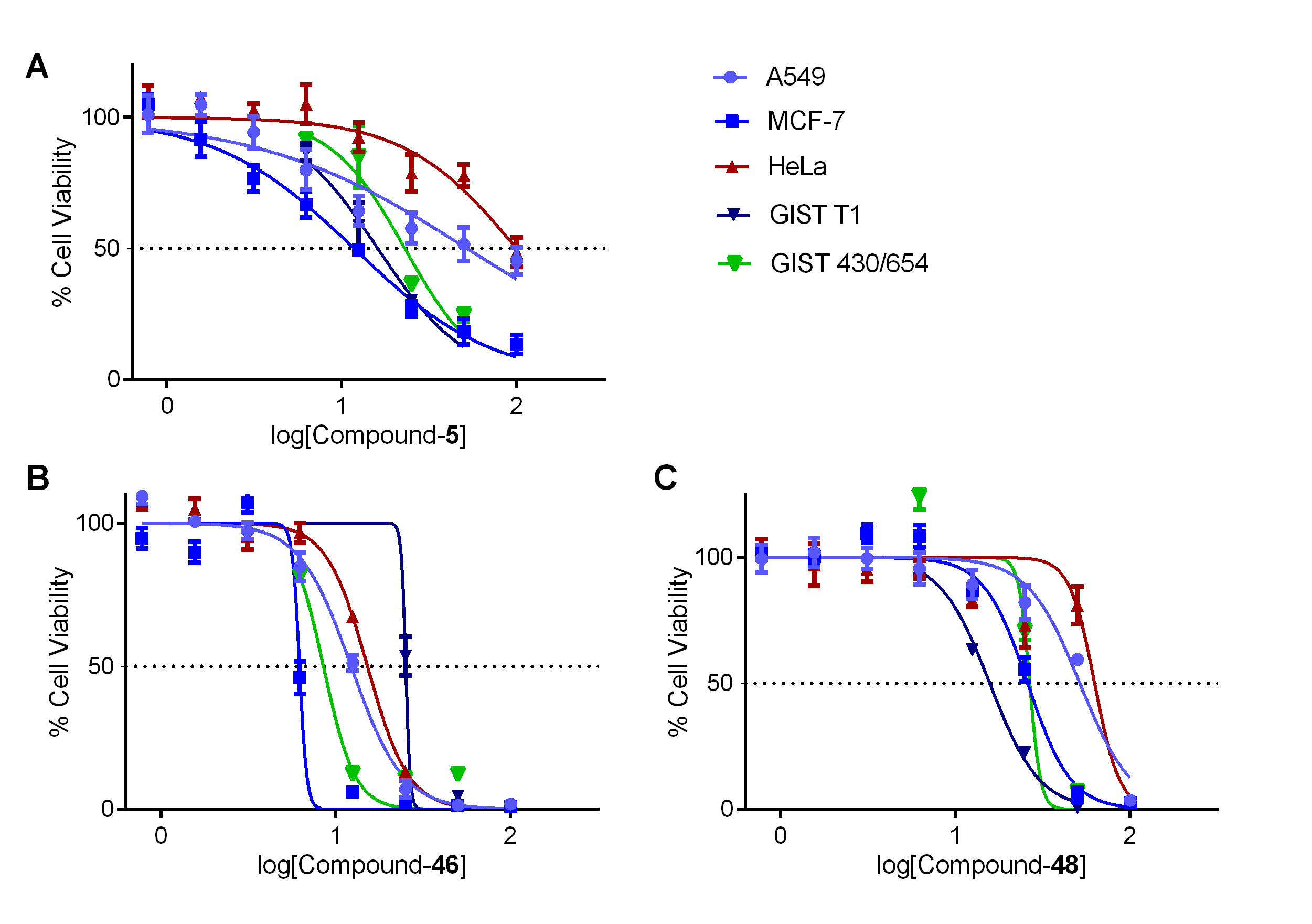

Supplement: Supplementary file 1 [file cancers-15-03817-s001.zip › Figure-10.png]

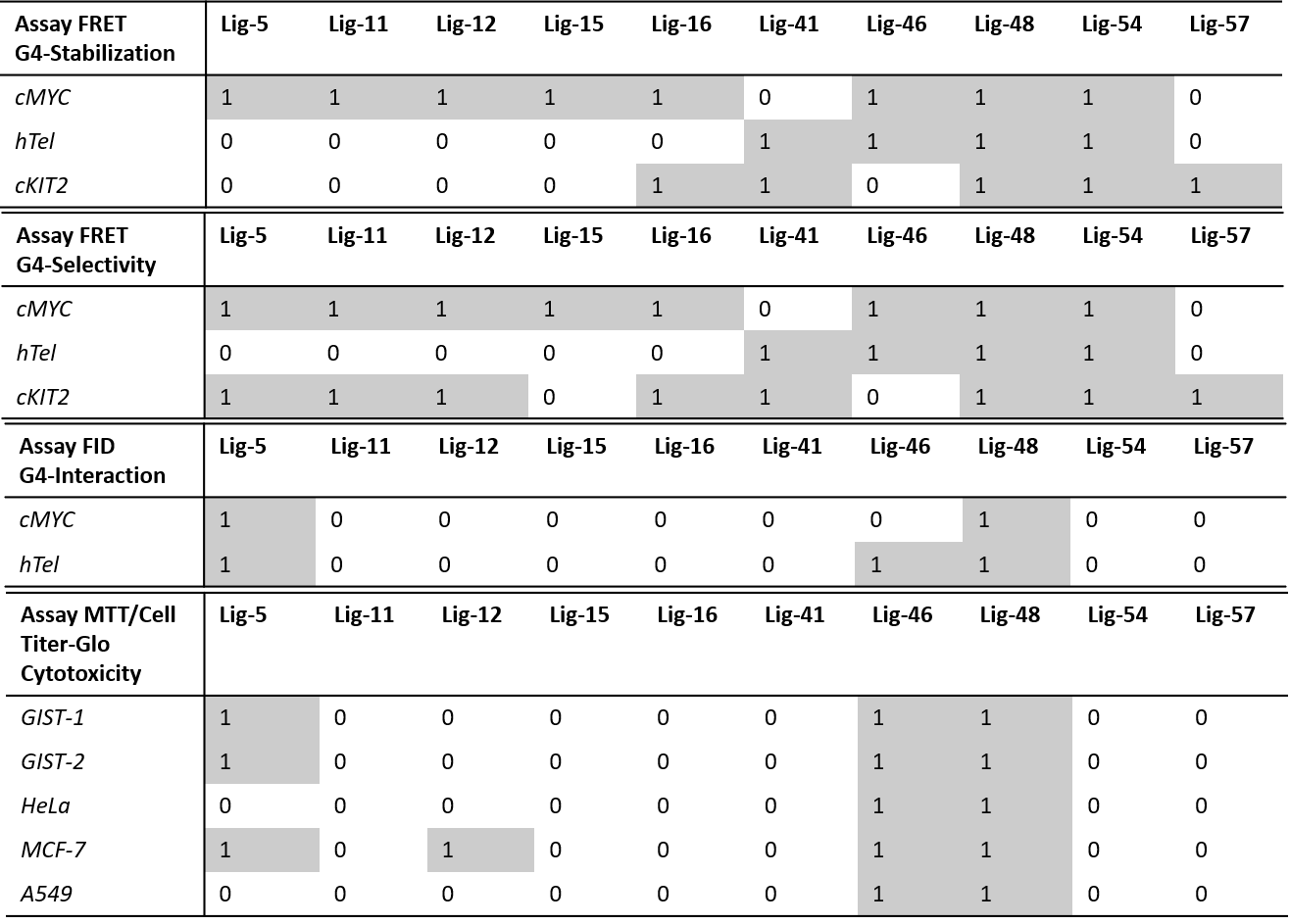

Supplement: Supplementary file 1 [file cancers-15-03817-s001.zip › Figure-11.png]

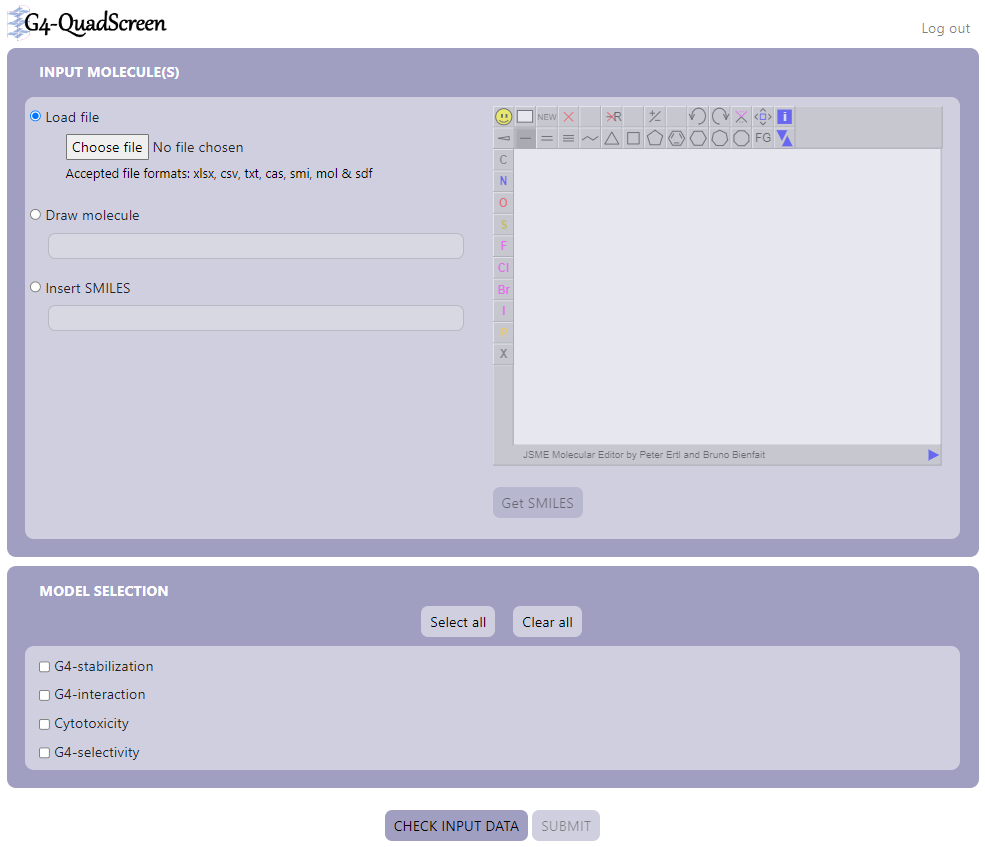

Supplement: Supplementary file 1 [file cancers-15-03817-s001.zip › Figure-5.png]

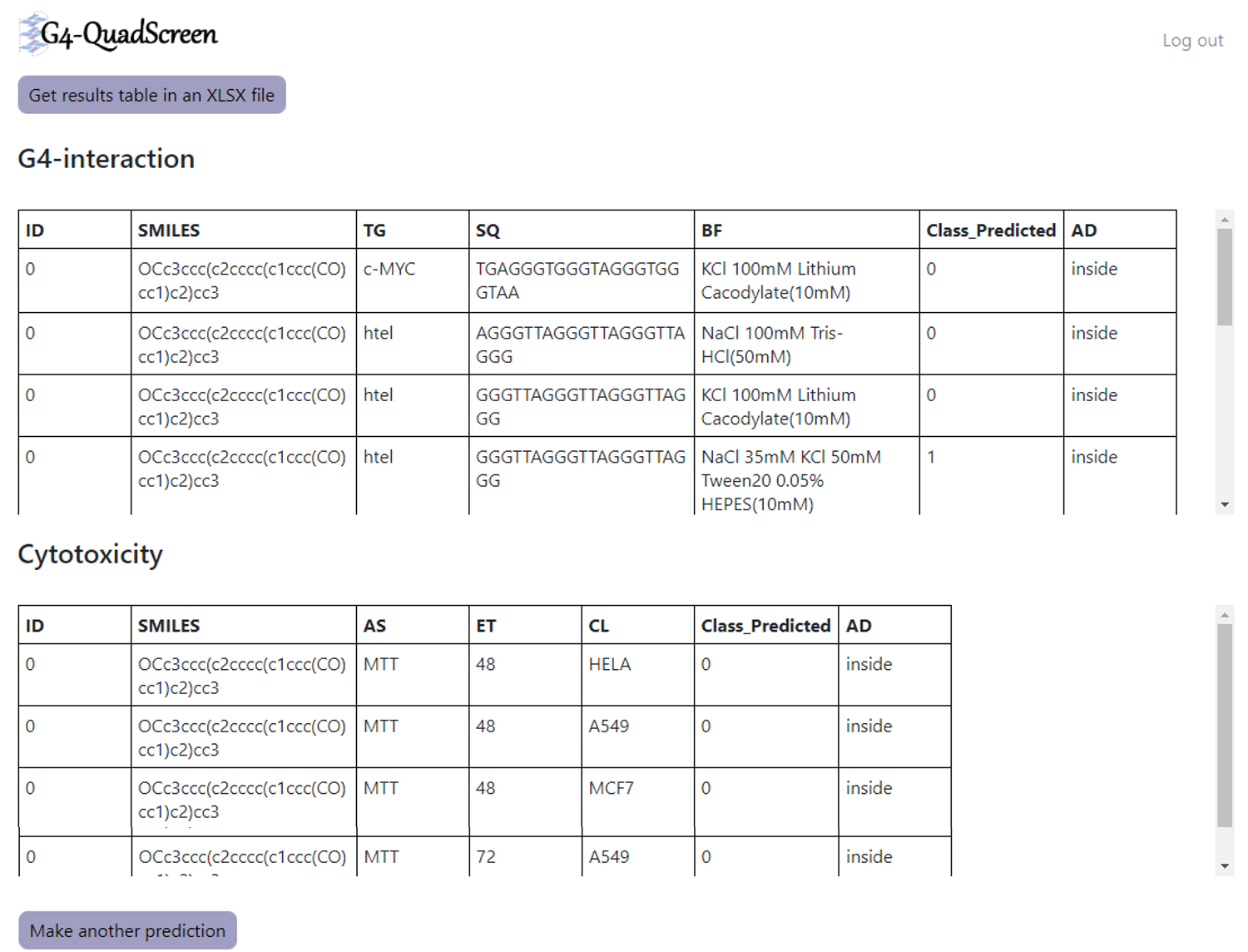

Supplement: Supplementary file 1 [file cancers-15-03817-s001.zip › Figure-6.png]

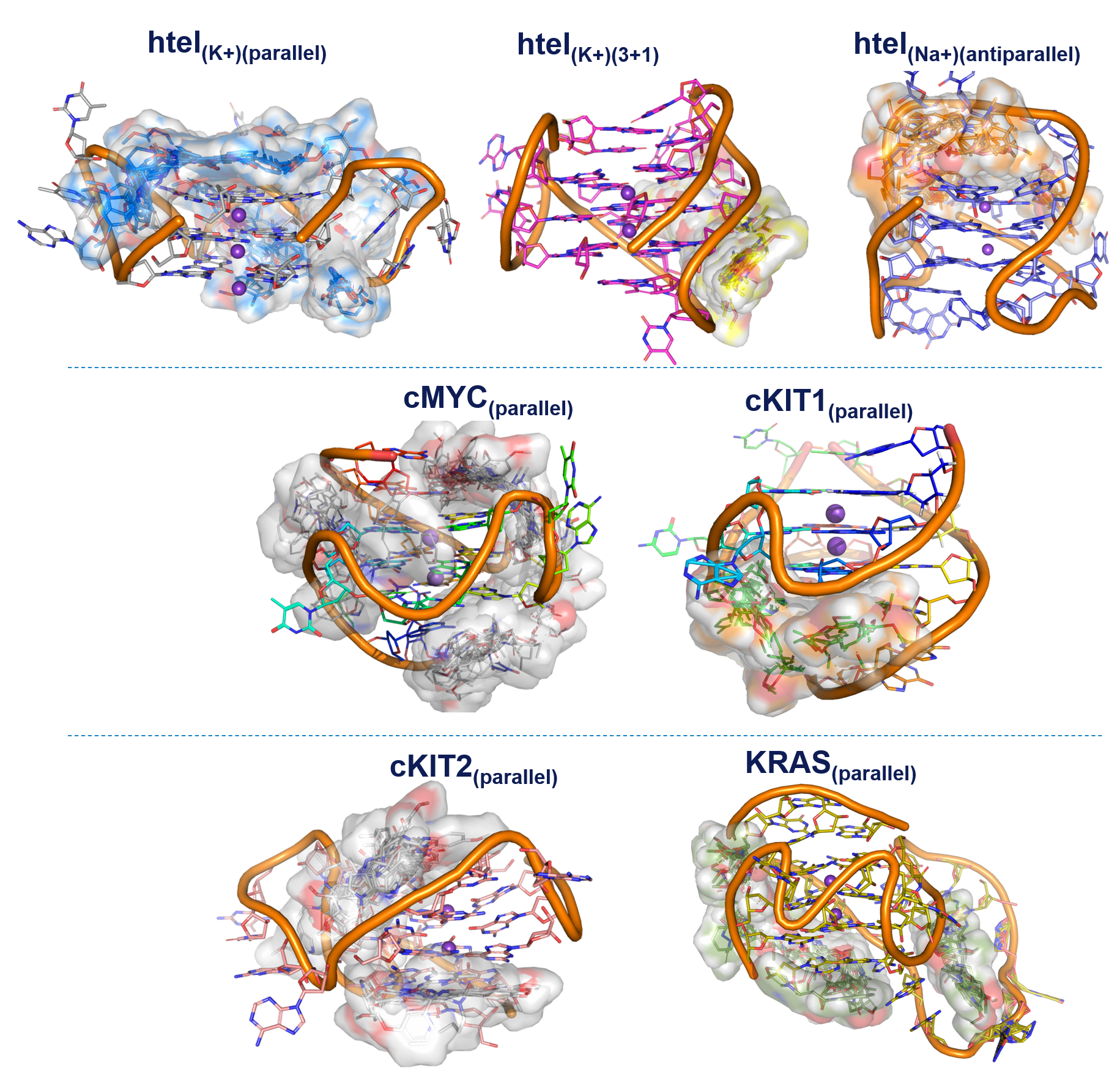

Supplement: Supplementary file 1 [file cancers-15-03817-s001.zip › Figure-7.png]

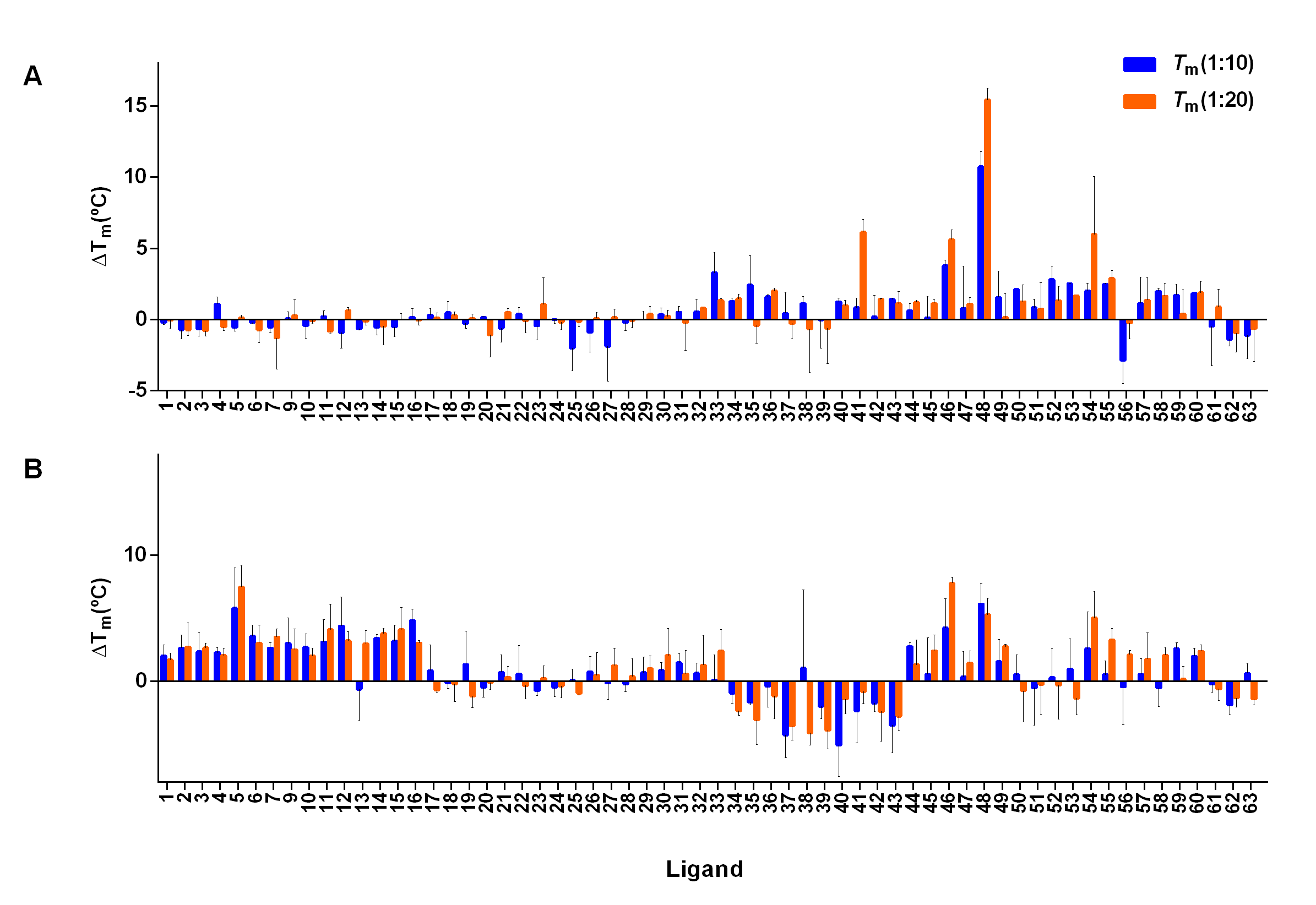

Supplement: Supplementary file 1 [file cancers-15-03817-s001.zip › Figure-8.png]

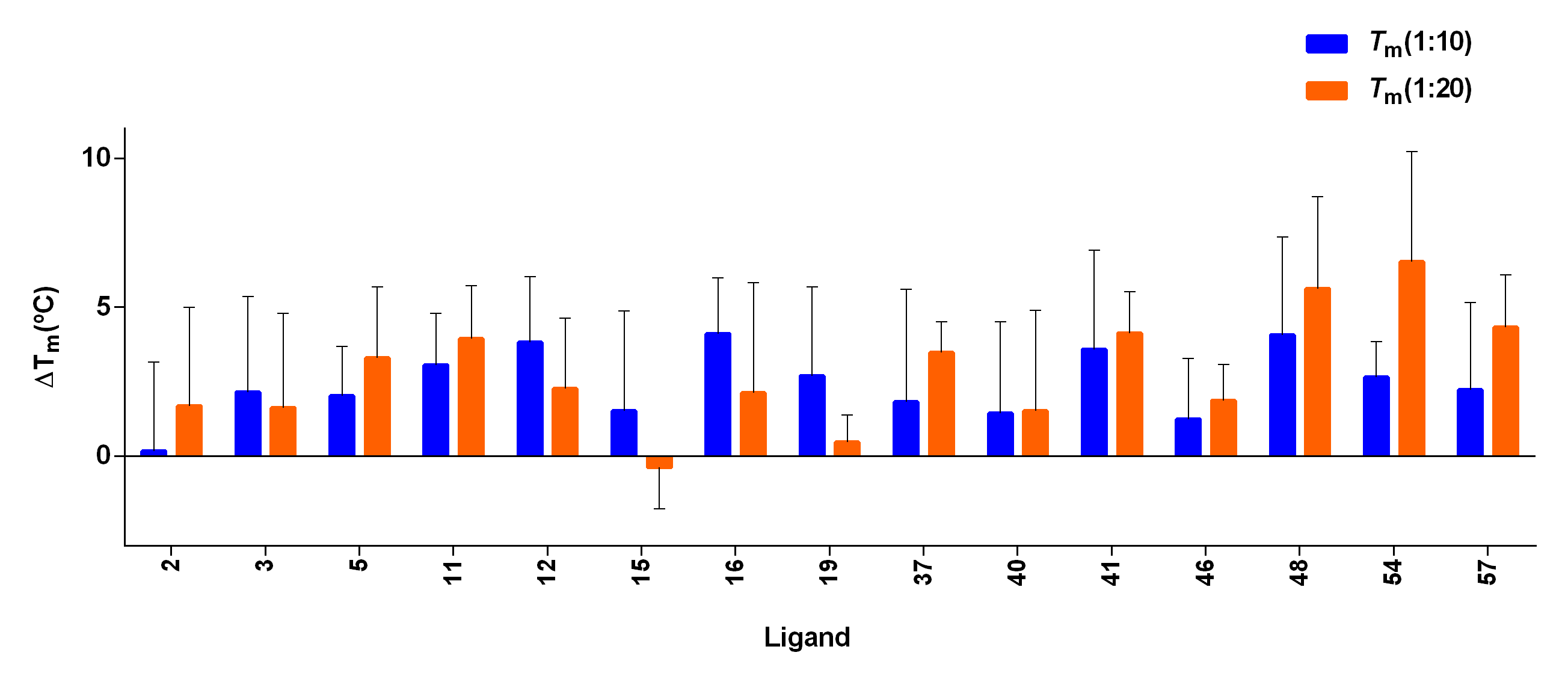

Supplement: Supplementary file 1 [file cancers-15-03817-s001.zip › Figure-9.png]

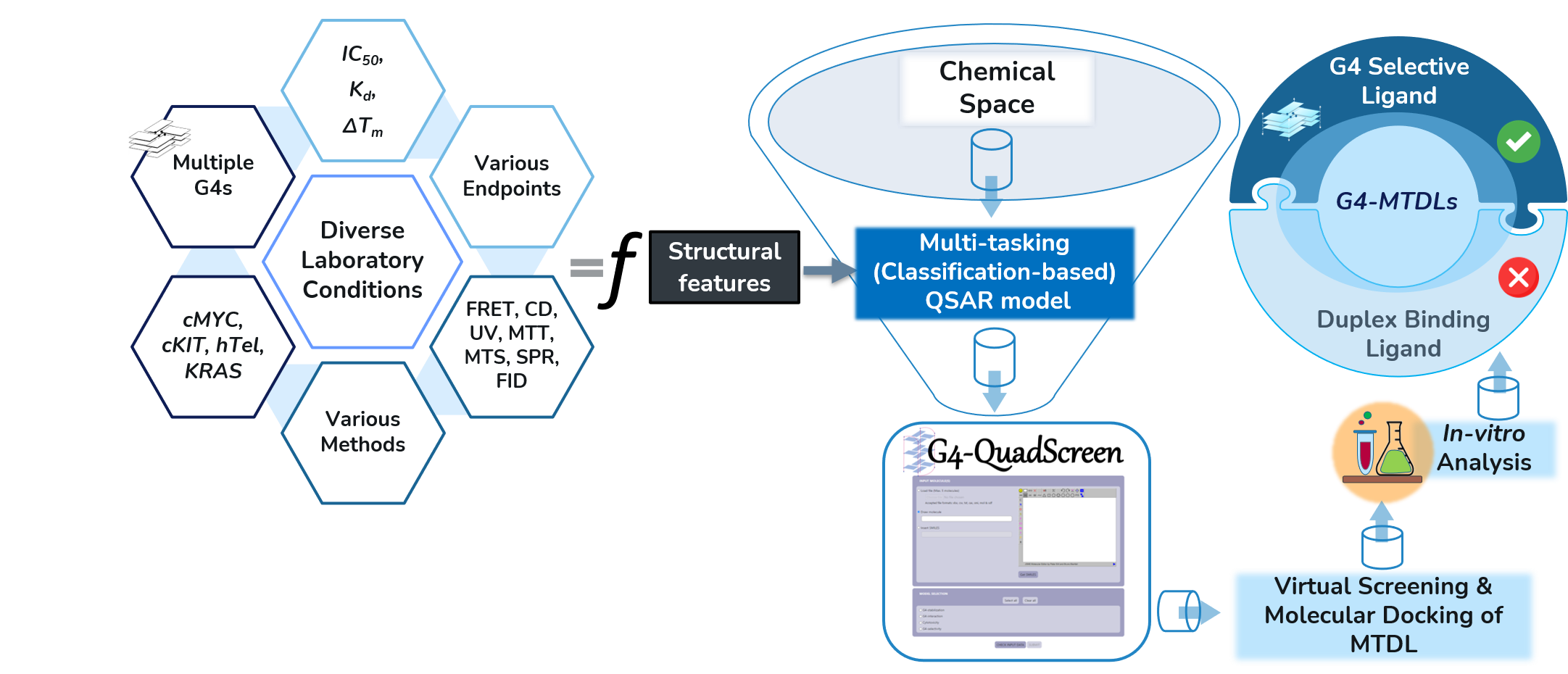

Supplement: Supplementary file 1 [file cancers-15-03817-s001.zip › Graphical-abstract.png]

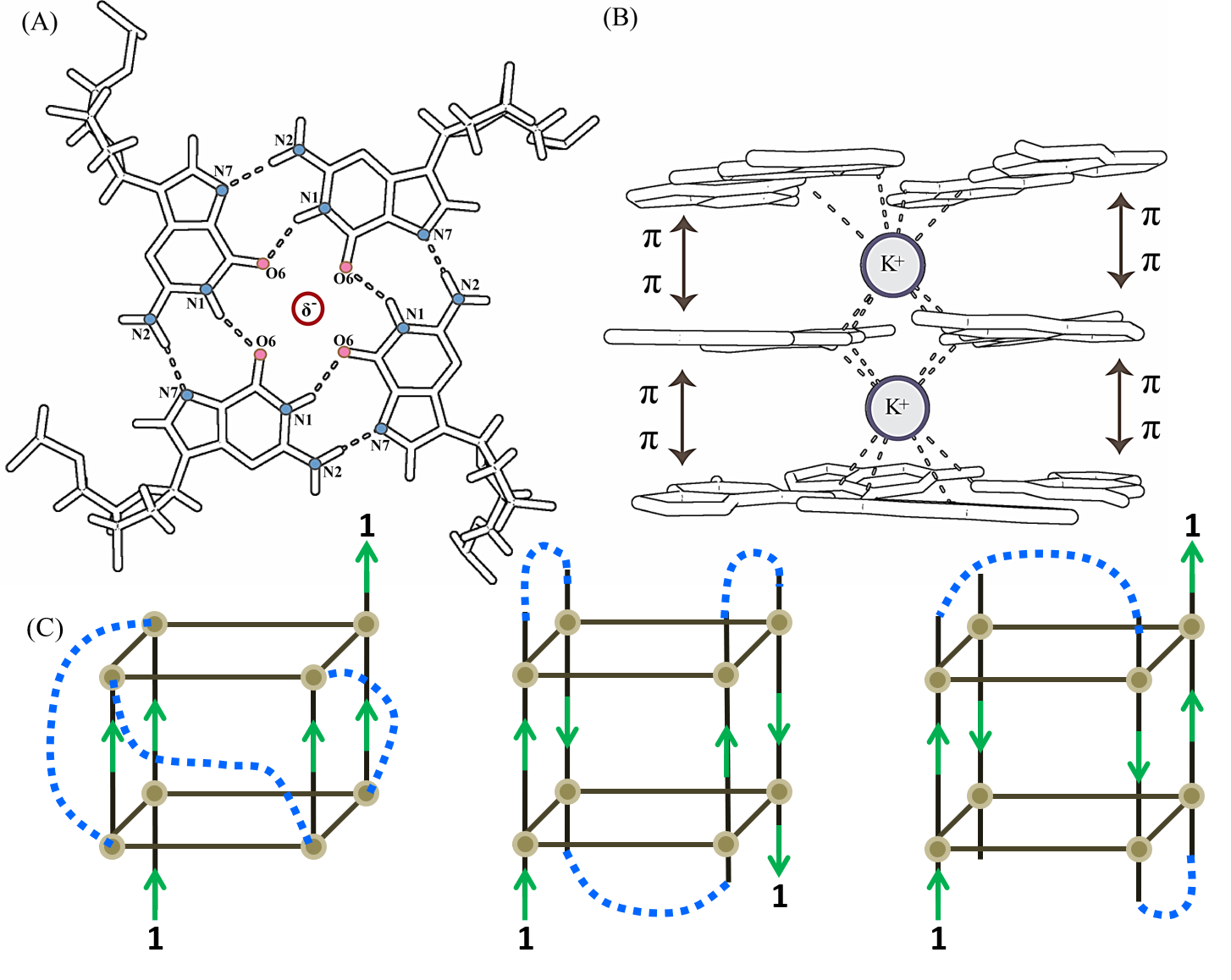

Supplement: Supplementary file 1 [file cancers-15-03817-s001.zip › Supplimentary/Figure-S1.png]

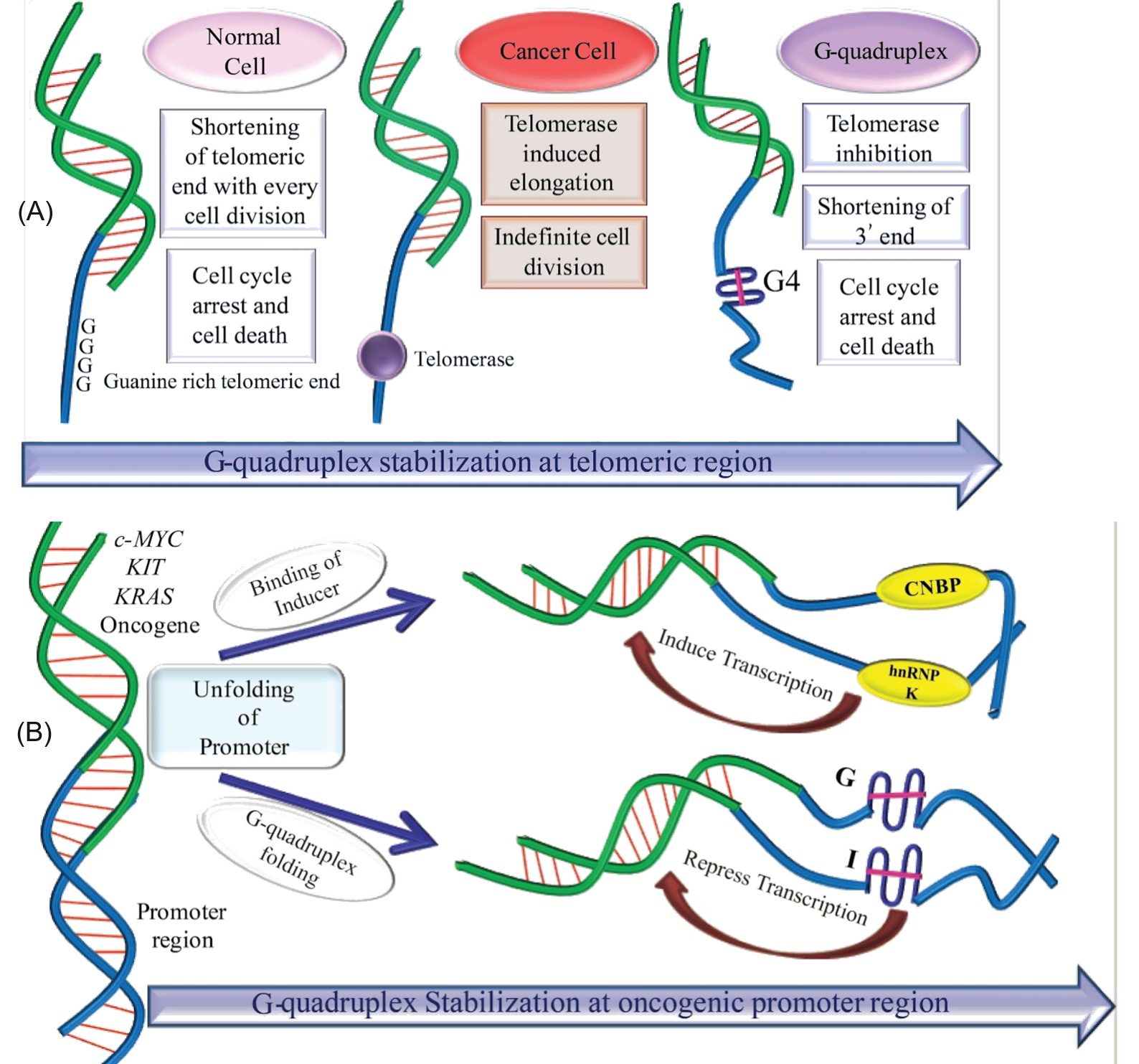

Supplement: Supplementary file 1 [file cancers-15-03817-s001.zip › Supplimentary/FIgure-S2.png]

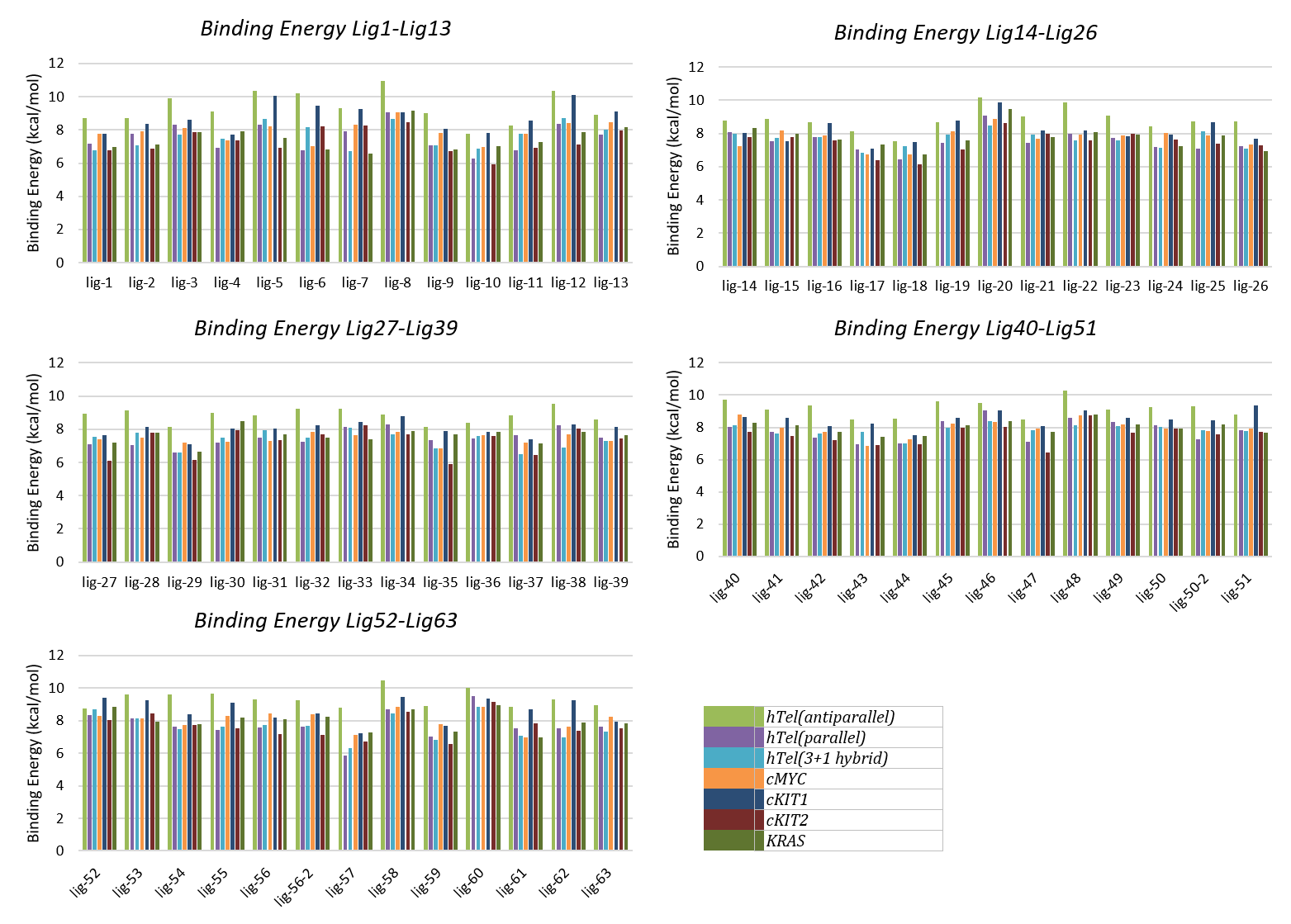

Supplement: Supplementary file 1 [file cancers-15-03817-s001.zip › Supplimentary/Figure-S3.png]

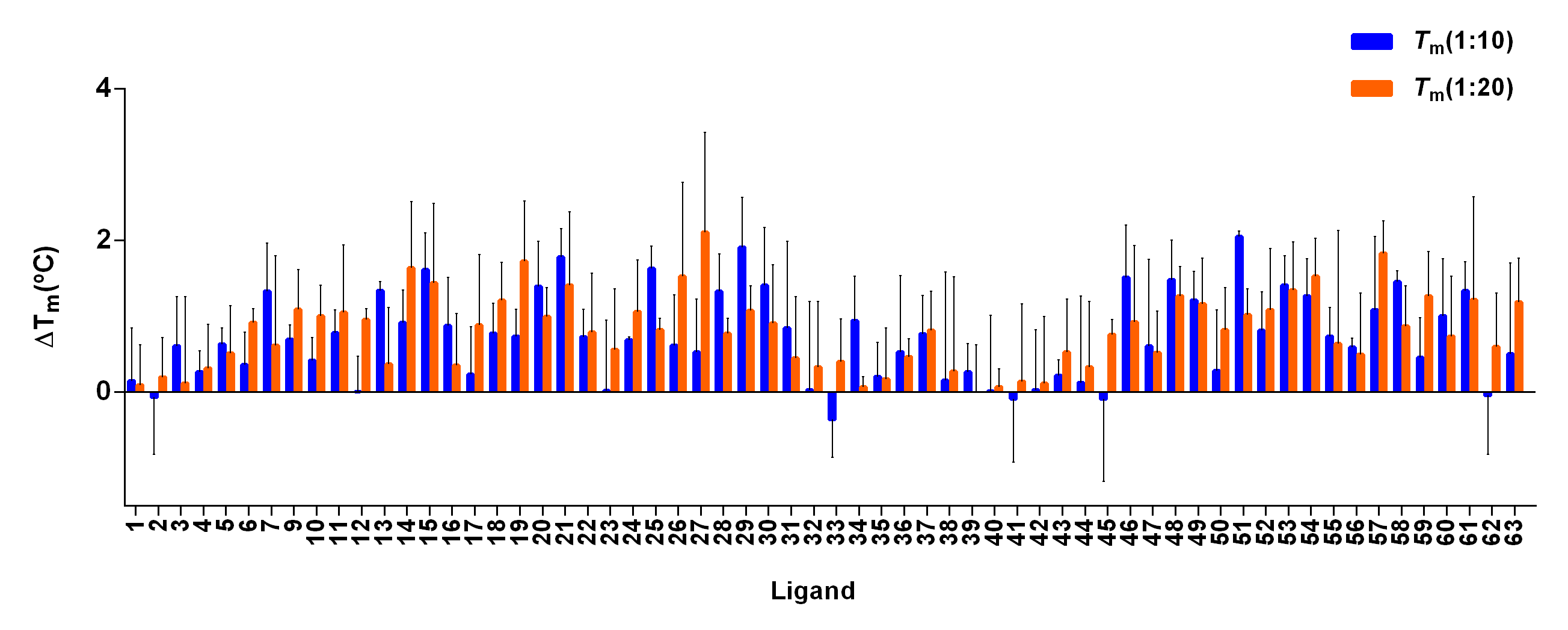

Supplement: Supplementary file 1 [file cancers-15-03817-s001.zip › Supplimentary/Figure-S4.png]

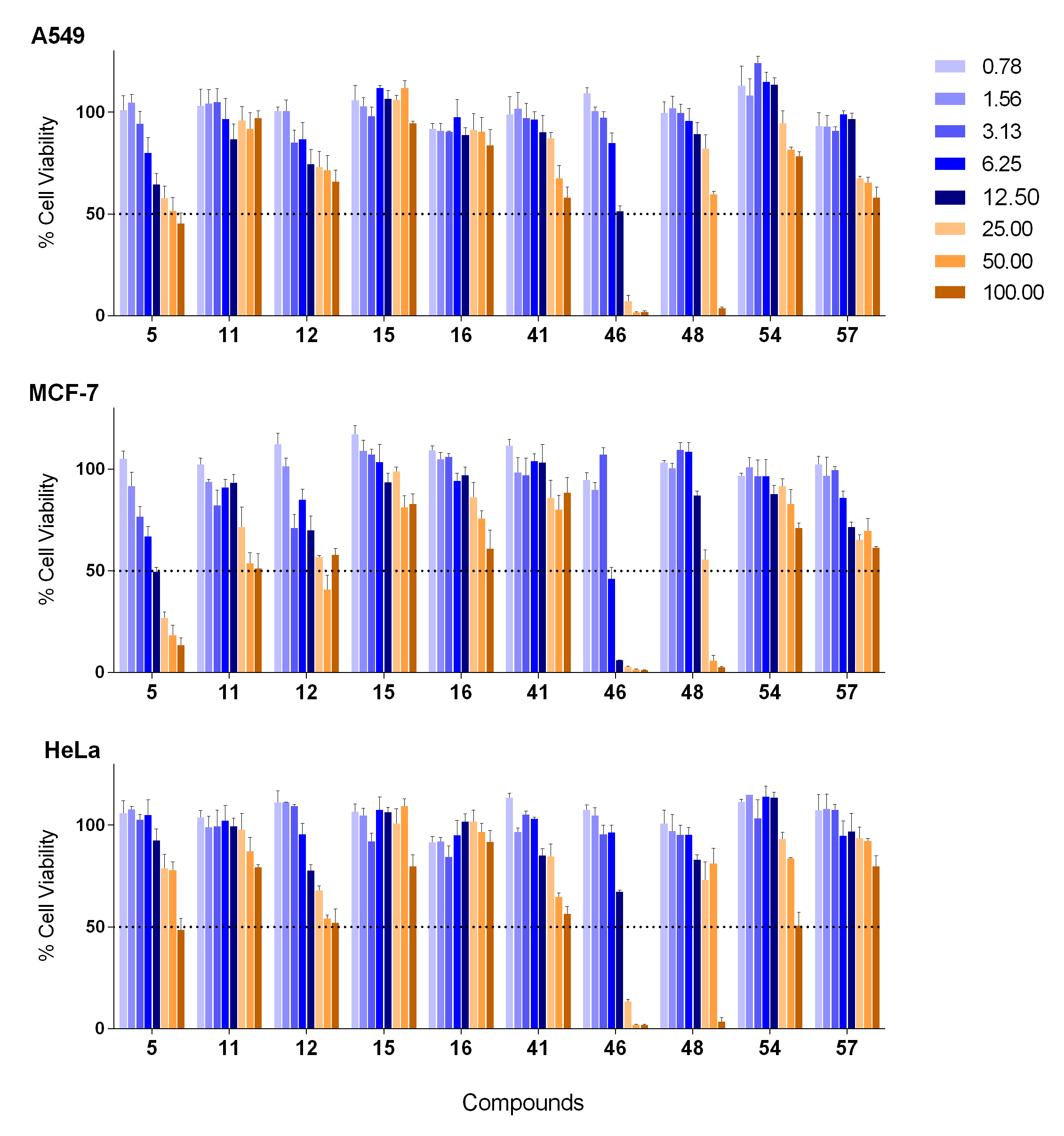

Supplement: Supplementary file 1 [file cancers-15-03817-s001.zip › Supplimentary/Figure-S5.png]

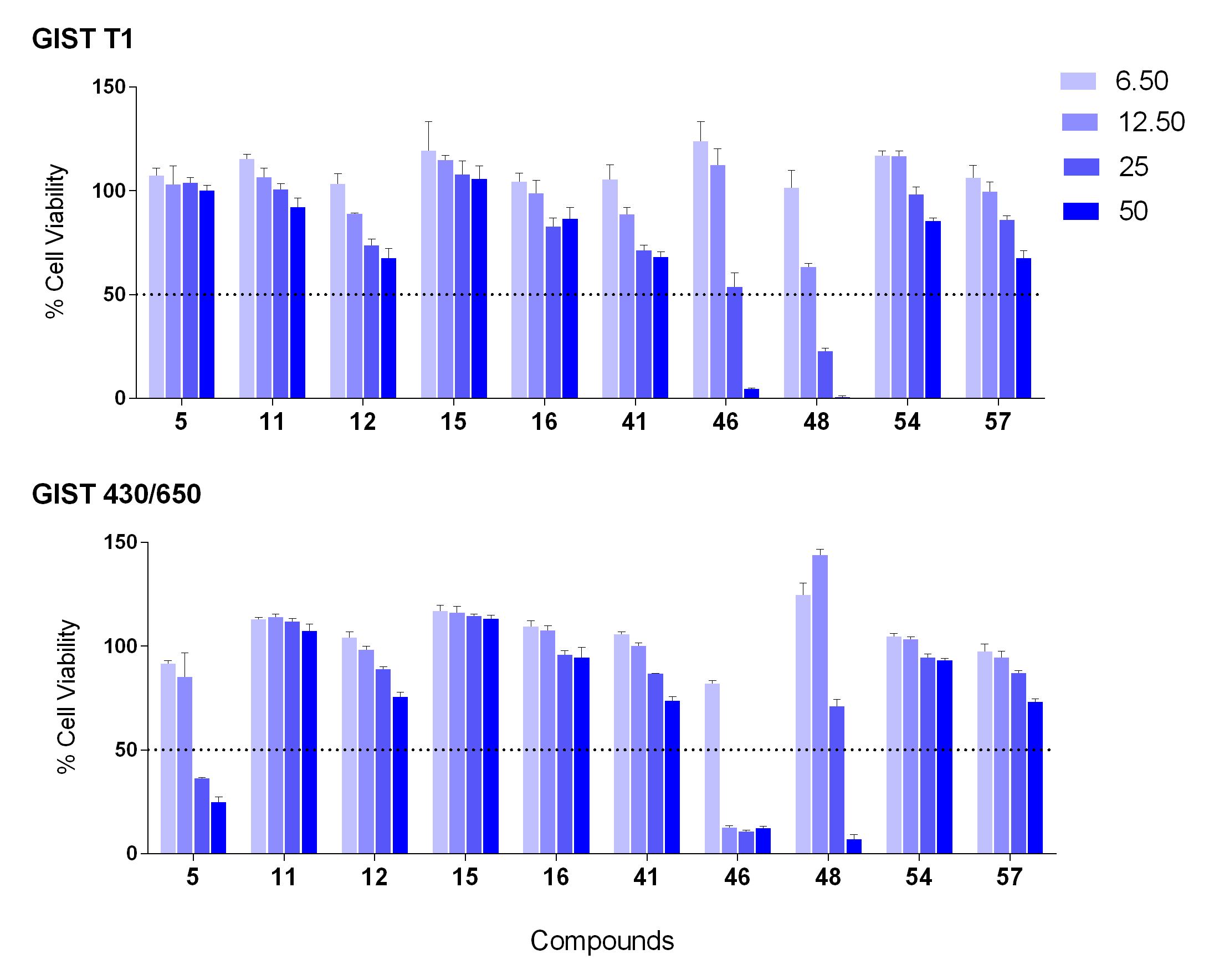

Supplement: Supplementary file 1 [file cancers-15-03817-s001.zip › Supplimentary/Figure-S6.png]

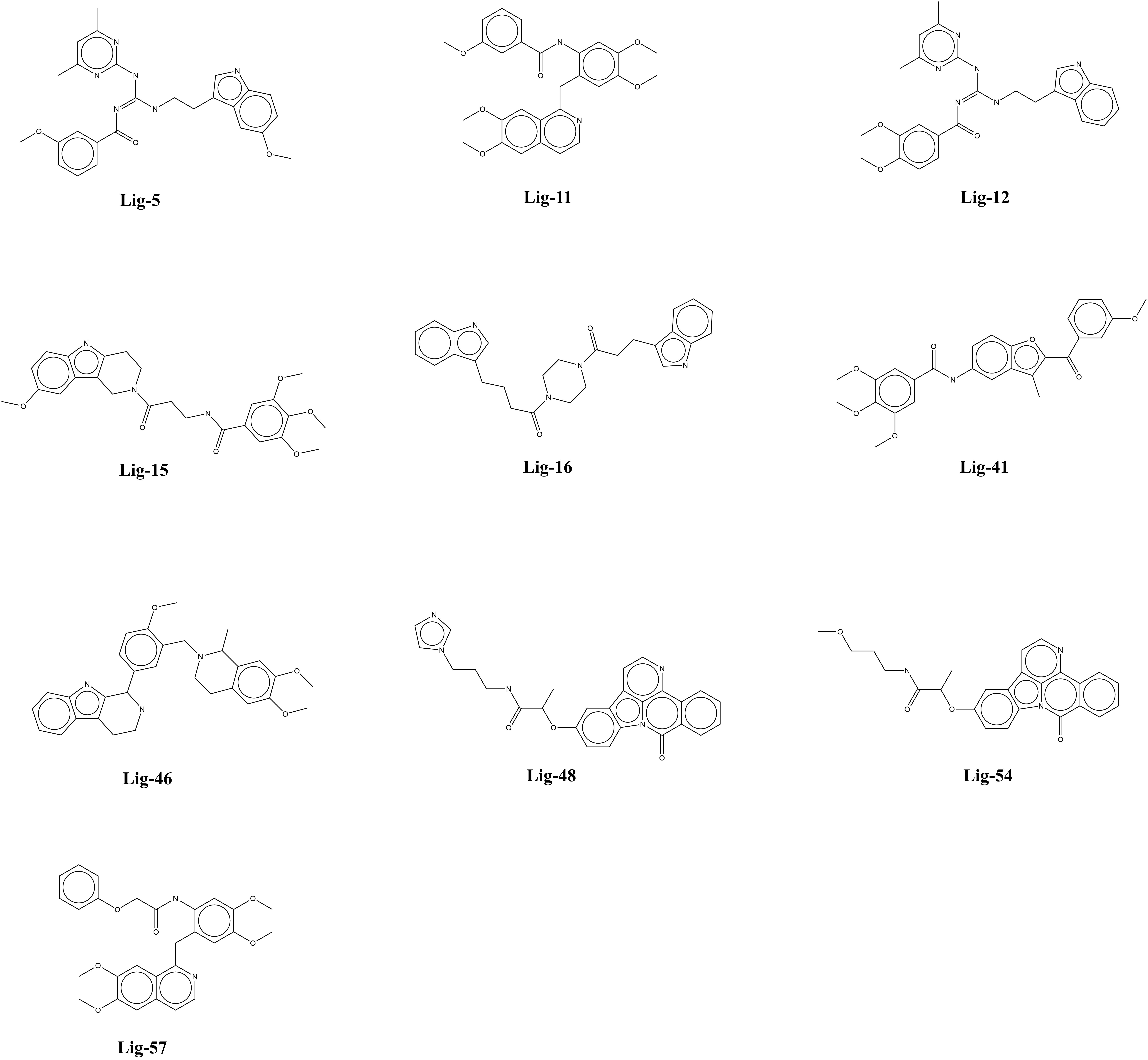

Supplement: Supplementary file 1 [file cancers-15-03817-s001.zip › Supplimentary/Figure-S7.png]
